# Heterogeneity between subgroups of first-line chemoimmunotherapy for extensive-stage small cell lung cancer patients: a meta-analysis and systematic review

**DOI:** 10.3389/fonc.2024.1334957

**Published:** 2024-10-18

**Authors:** Wenwen Kang, Jing Cheng, Luyun Pan, Ping Zhan, Hongbing Liu, Tangfeng Lv, Hedong Han, Yong Song

**Affiliations:** Jinling Hospital, Affiliated Hospital of Medical School, Nanjing University, Nanjing, China

**Keywords:** ES-SCLC, therapeutic heterogeneity, subgroup analysis, first-line chemoimmunotherapy, deft method

## Abstract

**Objectives:**

Differences in clinicopathological characteristics of extensive-stage small cell lung cancer (ES-SCLC) patients may influence the immune response. This study aims to evaluate the heterogeneity of response to first-line chemoimmunotherapy between subgroups in ES-SCLC to screen out suitable populations.

**Materials and methods:**

We searched the PubMed, EMBASE, and Cochrane Library databases from inception to December 3, 2022 for randomized controlled trials (RCTs) of ES-SCLC chemoimmunotherapy. We also reviewed main conferences from January 1, 2021 to October 1, 2023. A trial-specific hazard ratio (HR) ratio for each subgroup was calculated, and these ratios were then pooled using the deft approach.

**Results:**

A total of 9 RCTs with 4099 patients were finally included. The pooled ratios were 0.92 (95% CI = 0.77 to 1.09) for OS-HRs and 0.79 (95% CI = 0.55 to 1.13) for PFS-HRs in women versus men. The pooled ratios of OS-HRs and PFS-HRs in patients with positive versus negative PD-L1 expression were 1.26 (95% CI = 0.91 to 1.73) and 1.08 (95% CI = 0.77 to 1.52), respectively. The pooled ratios of OS-HRs and PFS-HRs in patients without versus with brain metastasis were 0.77 (95% CI = 0.59 to 1.01) and 0.71 (95% CI = 0.44 to 1.12). No statistically significant differences were also found in terms of subgroups for age, liver metastasis, smoking status, ECOG PS, LDH level, type of platinum salt and race.

**Conclusion:**

Women or patients with negative PD-L1 expression or with LDH ≤ ULN or without brain metastasis tend to benefit more from first-line chemoimmunotherapy in ES-SCLC. More trials are needed to prospectively validate the therapeutic heterogeneity among clinicopathological characteristics.

**Systematic review registration:**

https://inplasy.com/inplasy-2023-3-0064/ identifier, INPLASY202330064.

## 1 Introduction

Lung cancer is among the most common malignant tumors, with small cell lung cancer (SCLC) accounting for approximately 15% of all cases (1). SCLC is an aggressive neuroendocrine tumor originating from bronchial epithelial cells, and about 60%-70% of patients already have distant metastasis at diagnosis (2). Over the past 30 years, chemotherapy and radiotherapy were the primary clinical treatments for extensive-stage small cell lung cancer (ES-SCLC) patients, whereas effective time of them is short, and local recurrence or distant metastasis will occur soon. Overall, the 5-year survival rate of ES-SCLC patients is less than 2% (3, 4). Thus, we urgently need new treatment options for this recalcitrant cancer.

Immune checkpoint inhibitors (ICIs) can interrupt the immune escape system of tumors, enhance anti-tumor immunity and ultimately improve patient survival (5). However, the application of ICIs alone as a first-line treatment for SCLC patients is unsatisfactory, likely due to the rapid progression of SCLC, potential immune escape mechanisms and high potential risk of not undergoing chemotherapy (6, 7). Fortunately, immunotherapy can reverse the resistance of tumor cells to chemotherapy and reduce the toxicity of chemotherapy, while chemotherapy can enhance the anti-tumor activity in coordination with immunotherapy by enhancing tumor cell immunogenicity, removing immunosuppression and regulating the immune response (8, 9). Currently, chemoimmunotherapy seems to be the better first-line treatment option for ES-SCLC patients with a growing accumulation of phase II and III clinical researches data. The CAPSTONE-1 trial demonstrated that adebrelimab plus chemotherapy significantly improved survival in ES-SCLC patients, further validating the results of programmed death-ligand 1 (PD-L1) inhibitors plus chemotherapy in IMpower133 trial and CASPIAN trial (10–12). The ASTRUM-005 trial was the first to show that programmed death-1 (PD-1) inhibitors plus chemotherapy can also significantly prolong the survival of ES-SCLC patients (13). Moreover, serplulimab has been granted Orphan-Drug Designation (ODD) by the United States Food and Drug Administration (FDA) for the treatment of SCLC. The results of the RATIONALE-312 trial, presented at the 2023 World Conference on Lung Cancer (WCLC), further confirmed that ES-SCLC patients can achieve better survival outcomes (14).

It is well established that responses to chemoimmunotherapy vary among individuals, and it remains unclear which patients are most suited for this treatment. For example, NSCLC patients with positive PD-L1 expression may derive greater benefit from immunotherapy compared to those with negative PD-L1 expression. In view of the differences in clinical characteristics that may affect the efficacy of chemoimmunotherapy, we conducted this meta-analysis to directly explore potential therapeutic heterogeneity between subgroups and select the dominant groups more suitable for first-line chemoimmunotherapy in ES-SCLC, so as to maximize the therapeutic efficacy.

## 2 Materials and methods

### 2.1 Literature search

Two researchers (Kang and Han) independently searched the PubMed, EMBASE, and Cochrane Library databases from inception to December 3, 2022. The search terms included “extensive-small cell lung cancer”, “chemoimmunotherapy”, “PD-1 Inhibitors”, “Pembrolizumab”, “Nivolumab”, “Serplulimab”, “Cemiplimab”, “PD-L1 Inhibitors”, “Atezolizumab”, “Durvalumab”, “Adebrelimab”, “Avelumab”, “CTLA-4 Inhibitors”, “Ipilimumab”, “Tremelimumab”, “randomized controlled trial” (**Supplementary Table 1**). We also reviewed main conferences from January 1, 2021 to October 1, 2023.

This meta-analysis was conducted under the guidelines of Preferred Reporting Items for Systematic Reviews and Meta-analyses (PRISMA) (15) and registered on the INPLASY website (registration number: INPLASY202330064, https://inplasy.com/inplasy-2023-3-0064/).

### 2.2 Inclusion and exclusion criteria

Trials meeting the following criteria were included (1): phase II or III RCTs in patients with histological diagnosis of unresectable or advanced ES-SCLC (2); compared chemoimmunotherapy with chemotherapy as the first-line treatment (3); reported detailed outcomes including overall survival (OS), progression-free survival (PFS), objective response rate (ORR), disease control rate (DCR), treatment related adverse events (TRAEs) of grade 3 or higher and discontinuation rate (DR) (4); published in English. These trials with the latest and most comprehensive data were included.

### 2.3 Study selection and data extraction

Data collected included: trial name, first author, year of publication, treatment regimen, number of participants, and outcomes of included trials. To evaluate the therapeutic heterogeneity between subgroups, we also extracted HR and 95% confidence interval (CI) of OS and PFS in the following predefined subgroups: gender, age, PD-L1 expression level, brain metastasis, liver metastasis, smoking status, Eastern Cooperative Oncology Group performance status (ECOG PS), lactate dehydrogenase (LDH) level, the type of platinum salt and race. PD-L1 expression ≥ 1% tumor cell (TC) or tumor-infiltrating immune cell (IC) and PD-L1 tumor cell proportion score (TPS) ≥ 1% were considered to be positive PD-L1 expression (16). Finally, 9 RCTs were included (10–14, 17–23). Two authors (Kang and Han) independently extracted data and resolved the discrepancies by consensus.

### 2.4 Quality assessment and bias assessment

Using the Cochrane bias risk assessment tool (24), two authors (Kang and Han) independently assessed the risk of bias in each trial (**Supplementary Figure 9**). Studies were rated as low (low risk in all fields), high (high risk in one or more fields), and unclear risk of bias (more than 3 fields indicated unclear risk). Funnel plots were used to examine the presence of publication bias in our meta-analysis (**Supplementary Figure 10**).

### 2.5 Statistical analysis

All analyses were performed using a random-effects model. The primary endpoint was therapeutic heterogeneity between subgroups, measured by specific ratio of HRs (e.g. ratio of HR in women to HR in men). To avoid the risk of ecological bias for RCTs, the specific ratio of HR was calculated for each RCT and then combined using the deft method (25). We further performed subgroup analysis to explore therapeutic heterogeneity among patients receiving different types of chemoimmunotherapy.

The Q test was used to evaluate the heterogeneity between studies, and the I^2^ statistics were also calculated to represent the percentage of the total observed variability due to heterogeneity (26, 27). Sensitivity analysis was performed using a “one study deletion” approach. All tests were two-sided, and the results were considered statistically significant when the P value was less than 0.05. All analyses were performed using R software (version 4.2.2).

## 3 Results

### 3.1 Literature search and study selection

**Figure 1:**
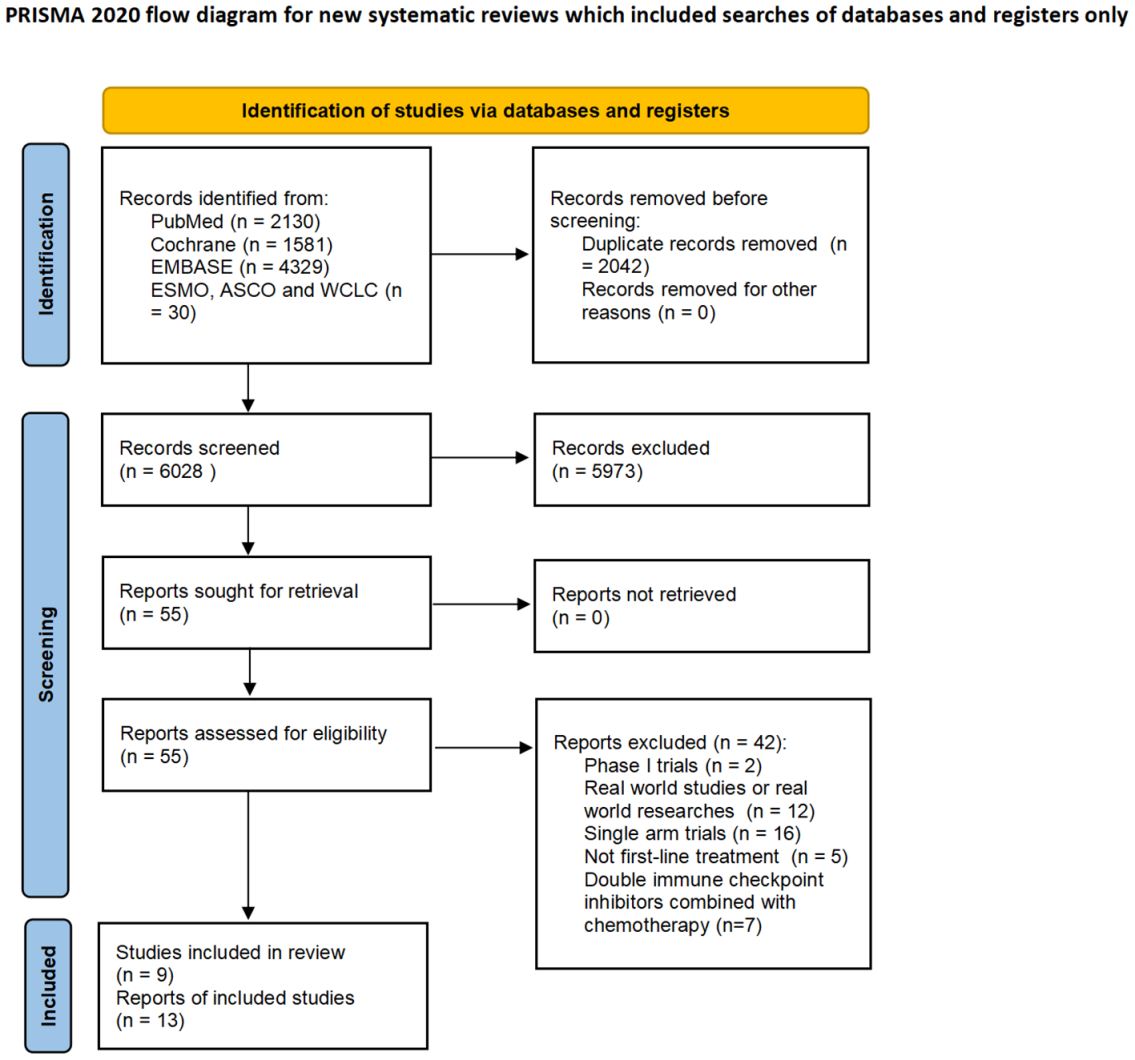
Flowchart of study selection and design.

8070 studies were identified on the initial literature search. A total of 9 RCTs with 4099 patients were finally included (**Figure 1**). The baseline characteristics of 9 RCTs were shown in **Table 1**, and patient characteristics across subgroups of trials were shown in **Table 2**. OS-HR data of subgroups were reported in 7 trials (**Supplementary Table 2**), and PFS-HR data of subgroups were reported in 4 trials (**Supplementary Table 3**).

**Table 1:** Main baseline characteristics of each included trial considered in this meta-analysis.

Trial	NCT number	Design	Experimental arm 1 (n)	Experimental arm 2 (n)	Control arm (n)
CA184-041	NCT00527735	A randomized, multicenter, double -blind, parallel, phase 2 trial	(n = 42) phase regman: Ipilimumab + CP	(n = 43) concurrent regman: Ipilimumab + CP	(n = 45) CP
CA184-156	NCT01450761	A randomized, multicenter, double-blind, parallel, phase 3 trial	(n = 478) Ipilimumab + EP		(n = 476) Placebo + EP
IMpower133	NCT02763579	A randomized, multicenter, double-blind, parallel, phase 3 trial	(n = 201) Atezolizumab + EP		(n = 202) Placebo + EP
EA5161	NCT03382561	A randomized, open-label, parallel, phase 2 trial	(n = 80) Nivolumab + EP		(n = 80) EP
KEYNOTE-604	NCT03066778	A randomized, multicenter, double-blind, parallel, phase 3 trial	(n = 228) Pembrolizumab + EP		(n = 225) Placebo + EP
CASPIAN	NCT03043872	A randomized, multicenter, open-label, parallel, phase 3 trial	(n = 268) Durvalumab + EP	(n = 268) Durvalumab + Tremelimumab + EP	(n = 269) EP
CAPSTONE-1	NCT03711305	A randomized, multicenter, double-blind, parallel, phase 3 trial	(n = 230) Adebrelimab + EP		(n = 232) Placebo + EP
ASTRUM-005	NCT04063163	A randomized, multicenter, double-blind, parallel, phase 3 trial	(n = 389) Serplulimab + EP		(n = 196) Placebo + EP
RATIONALE-312	NCT04005716	A randomized, multicenter, double-blind, parallel, phase 3 trial	(n = 227) Tislelizumab + EP		(n = 230) Placebo + EP

**Table 2:** Patient characteristics of each included trial considered in this meta-analysis.

Patient characteristics		CA184-041		CA184-156		IMpower133		CASPIAN		CAPSTONE-1		EA5161		KEYNOTE-604		ASTRUM-005		RATIONALE-312	
		CT+IO n=43 (%)	CT n=45 (%)	CT+IO n=478 (%)	CT+PLA n=476 (%)	CT+IO n=201 (%)	CT+PLA n=202 (%)	CT+IO n=268 (%)	CT n=269 (%)	CT+IO n=230 (%)	CT+PLA n=232 (%)	CT+IO n=80 (%)	CT n=80 (%)	CT+IO n=228 (%)	CT+PLA n=225 (%)	CT+IO n=389 (%)	CT+PLA n=196 (%)	CT+IO n=227 (%)	CT+PLA n=230 (%)
Sex	Male	33 (76.7)	33 (73.3)	317 (66.3)	326 (68.5)	129 (64.2)	132 (65.3)	190 (70.9)	184 (68.4)	184 (80.0)	188 (81.0)	35 (43.7)	36 (45.0)	152 (66.7)	142 (63.1)	317 (81.5)	164 (83.7)	186 (81.9)	186 (80.9)
	Female	10 (23.3)	12 (26.7)	161 (33.7)	150 (31.5)	72 (35.8)	70 (34.7)	78 (29.1)	85 (31.6)	46 (20.0)	44 (19.0)	45 (56.3)	44 (55.0)	76 (33.3)	83 (36.9)	72 (18.5)	32 (16.3)	41 (18.1)	44 (19.1)
Age	<65	35 (81.4)	36 (80.0)	299 (62.6)	277 (58.2)	111 (55.2)	106 (52.5)	167 (62.3)	157 (58.4)	155 (67.4)	147 (63.4)	NA	NA	115 (50.4)	101 (44.9)	235 (60.4)	119 (60.7)	138 (60.8)	149 (64.8)
	≥65	8 (18.6)	9 (20.0)	179 (37.4)	199 (41.8)	90 (44.8)	96 (47.5)	101 (37.7)	112 (41.6)	75 (32.6)	85 (36.6)	NA	NA	113 (49.6)	124 (55.1)	154 (39.6)	77 (39.3)	89 (39.2)	81 (35.2)
Race	Asian	NA	NA	108 (22.6)	107 (22.5)	33 (16.4)	36 (17.8)	36 (13.4)	42 (15.6)	230 (100)	232 (100)	NA	NA	52 (22.8)	32 (14.2)	262 (67.4)	139 (70.9)	NA	NA
	Non-Asian	NA	NA	370 (77.4)	369 (77.5)	168 (83.6)	166 (82.2)	232 (86.6)	227 (84.4)	0 (0)	0 (0)	NA	NA	176 (77.2)	193 (85.8)	127 (32.6)	57 (29.1)	NA	NA
ECOG PS	0	8 (18.6)	12 (26.7)	137 (28.7)	147 (30.9)	73 (36.3)	67 (33.2)	99 (36.9)	90 (33.5)	33 (14.3)	30 (12.9)	23 (28.7)	24 (30.0)	60 (26.3)	56 (24.9)	71 (18.3)	32 (16.3)	35 (15.4)	34 (14.8)
	1	34 (79.1)	33 (73.3)	340 (71.1)	328 (68.9)	128 (63.7)	135 (66.8)	169 (63.1)	179 (66.5)	197 (85.7)	202 (87.1)	57 (71.3)	56 (70.0)	168 (73.7)	169 (75.1)	318 (81.7)	164 (83.7)	192 (84.6)	196 (85.2)
Platinum salt	Carboplatin	43 (100)	45 (100)	314 (65.7)	317 (66.6)	201 (100)	202 (100)	201 (75.0)	201 (74.7)	230 (100)	232 (100)	NA	NA	161 (70.6)	156 (69.3)	389 (100)	196 (100)	180 (79.3)	181 (78.7)
	Cisplatin	0 (0)	0 (0)	164 (34.3)	159 (33.4)	0 (0)	0 (0)	67 (25.0)	68 (25.3)	0 (0)	0 (0)	NA	NA	67 (29.4)	69 (30.7)	0 (0)	0 (0)	47 (20.7)	49 (21.3)
Brain mts	Yes	0 (0)	0 (0)	55 (11.5)	45 (9.5)	17 (8.5)	18 (8.9)	28 (10.4)	27 (10.0)	5 (2.2)	5 (2.2)	NA	NA	33 (14.5)	22 (9.8)	50 (12.9)	28 (14.3)	1 (0.4)	4 (1.7)
	No	43 (100)	45 (100)	423 (88.5)	431 (90.5)	184 (91.5)	184 (91.1)	240 (89.6)	242 (90.0)	225 (97.8)	227 (97.8)	NA	NA	195 (85.5)	203 (90.2)	339 (87.1)	168 (85.7)	226 (99.6)	226 (98.3)
Liver mts	Yes	NA	NA	NA	NA	77 (38.3)	72 (35.6)	108 (40.3)	104 (38.7)	73 (31.7)	74 (31.9)	NA	NA	95 (41.7)	92 (40.9)	99 (25.4)	51 (26.0)	64 (28.2)	59 (25.7)
	No	NA	NA	NA	NA	124 (61.7)	130 (63.4)	160 (59.7)	165 (61.3)	157 (68.3)	158 (68.1)	NA	NA	133 (58.3)	133 (59.1)	290 (74.6)	145 (74.0)	163 (71.8)	171 (74.3)
Smoking status	Smoker	38 (88.4)	41 (91.1)	268 (56.1)	271 (56.9)	192 (95.5)	199 (98.5)	246 (91.8)	254 (94.4)	180 (78.3)	179 (77.2)	NA	NA	220 (96.5)	217 (96.4)	308 (79.2)	161 (82.1)	174 (76.7)	171 (74.3)
	Non-Smoker	5 (11.6)	4 (8.9)	172 (36.0)	167 (35.1)	9 (4.5)	3 (1.5)	22 (8.2)	15 (5.6)	50 (21.7)	53 (22.8)	NA	NA	8 (3.5)	8 (3.6)	81 (20.8)	35 (17.9)	53 (23.3)	59 (25.7)
LDH level	≤ULN	NA	NA	242 (50.6)	246 (51.7)	NA	NA	NA	NA	116 (50.4)	115 (49.6)	NA	NA	100 (43.9)	95 (42.2)	NA	NA	114 (50.2)	109 (47.4)
	>ULN	25 (58.1)	19 (42.2)	231 (48.3)	228 (47.9)	NA	NA	NA	NA	114 (49.6)	117 (50.4)	NA	NA	127 (55.7)	129 (57.3)	NA	NA	113 (49.8)	121 (52.6)
PD-L1 expression level	<1%	NA	NA	NA	NA	36/64 (56.3)	36/73 (49.3)	NA	NA	196 (85.2)	200 (86.2)	NA	NA	97 (42.5)	78 (34.7)	317 (81.5)	152 (77.6)	NA	NA
	≥1%	NA	NA	NA	NA	28/64 (43.8)	37/73 (50.7)	NA	NA	24 (10.4)	20 (8.6)	NA	NA	88 (38.6)	97 (43.1)	62 (15.9)	34 (17.3)	NA	NA
	Unknow	NA	NA	NA	NA	NA	NA	NA	NA	10 (4.3)	12 (5.2)	NA	NA	43 (18.9)	50 (22.2)	10 (2.6)	10 (5.1)	NA	NA

CT, platinum-based chemotherapy; IO, immune-oncology; PLA, placebo; PD-L1, programmed death-ligand 1; ECOG PS, Eastern Cooperative Oncology Group Performance Status; LDH, lactate dehydrogenase; ULN, upper limit of normal; mts, metastases; NA, not available.

### 3.2 Comparison of the efficacy of chemoimmunotherapy versus chemotherapy as the first-line treatment of ES-SCLC patients

**Figure 2:**
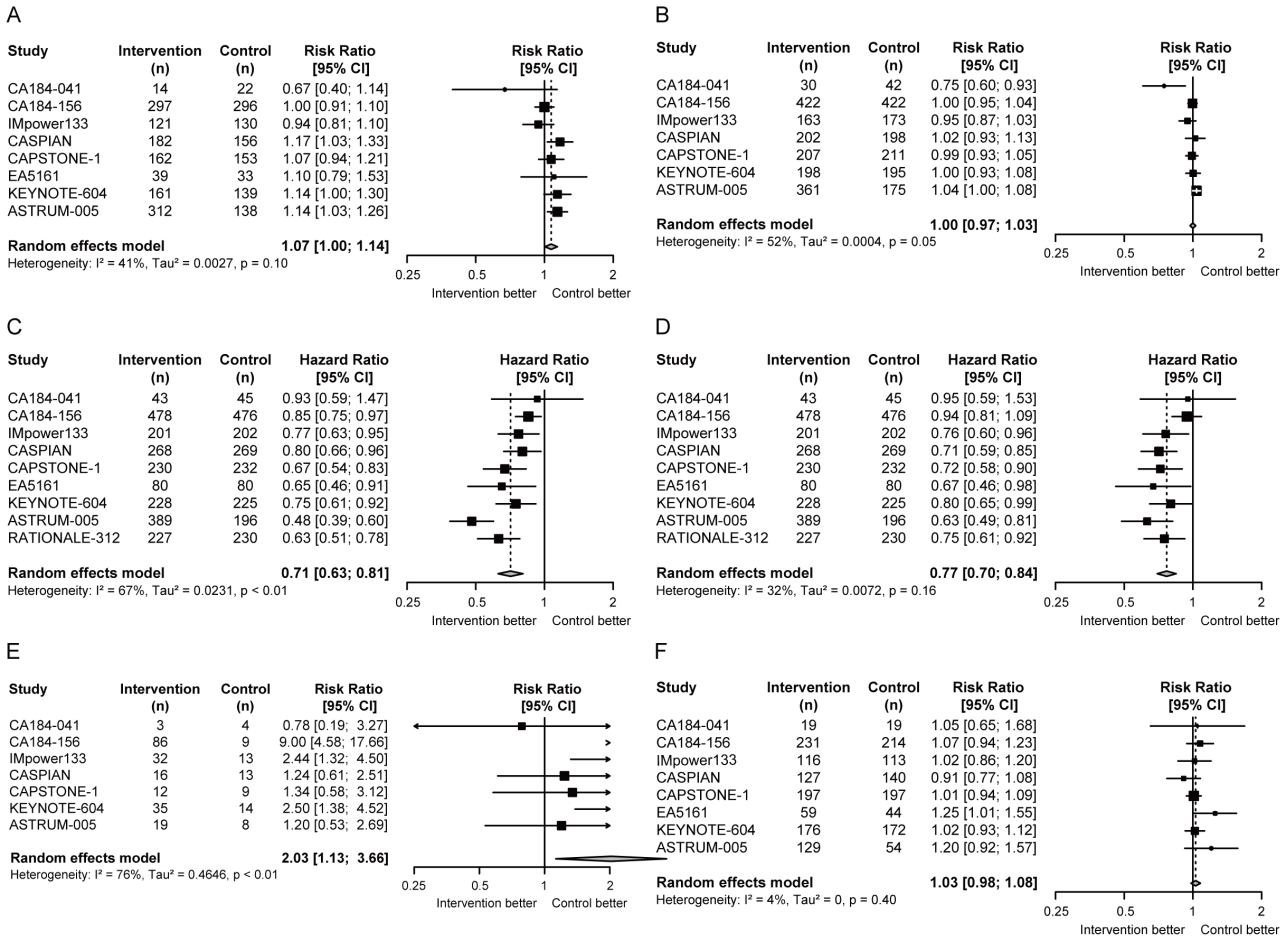
Forest plots of efficacy and safety endpoints in ES-SCLC patients receiving first-line chemoimmunotherapy versus chemotherapy. **(A)** RRs of ORR; **(B)** RRs of DCR; **(C)** HR of PFS; **(D)** HR of OS; **(E)** RRs of DR; **(F)** RRs of TRAEs. ORR, objective response rate; DCR, disease control rate; PFS, progression-free survival; OS, overall survival; DR, discontinuation rate; TRAEs, treatment-related adverse events; RR, risk ratio; HR, hazard ratio; CI, confidence interval.

Compared with chemotherapy alone, no obvious advantages of chemoimmunotherapy were observed in ORR (RR = 1.07, 95% CI = 1.00 to 1.14; **Figure 2A**) and DCR (RR = 1.00, 95% CI = 0.97 to 1.03; **Figure 2B**). Notably, PFS (HR = 0.71, 95% CI = 0.63 to 0.81; **Figure 2C**) and OS (HR = 0.77, 95% CI = 0.70 to 0.84; **Figure 2D**) were significantly prolonged in ES-SCLC patients receiving chemoimmunotherapy. As for the safety of chemoimmunotherapy, it resulted in an increase in DR (RR = 2.03, 95% CI = 1.13 to 3.66; **Figure 2E**), but no statistically significant increase in TRAEs (RR = 1.03, 95% CI = 0.98 to 1.08; **Figure 2F**).

### 3.3 Heterogeneity between subgroups of chemoimmunotherapy as the first-line treatment of ES-SCLC patients

**Figure 3:**
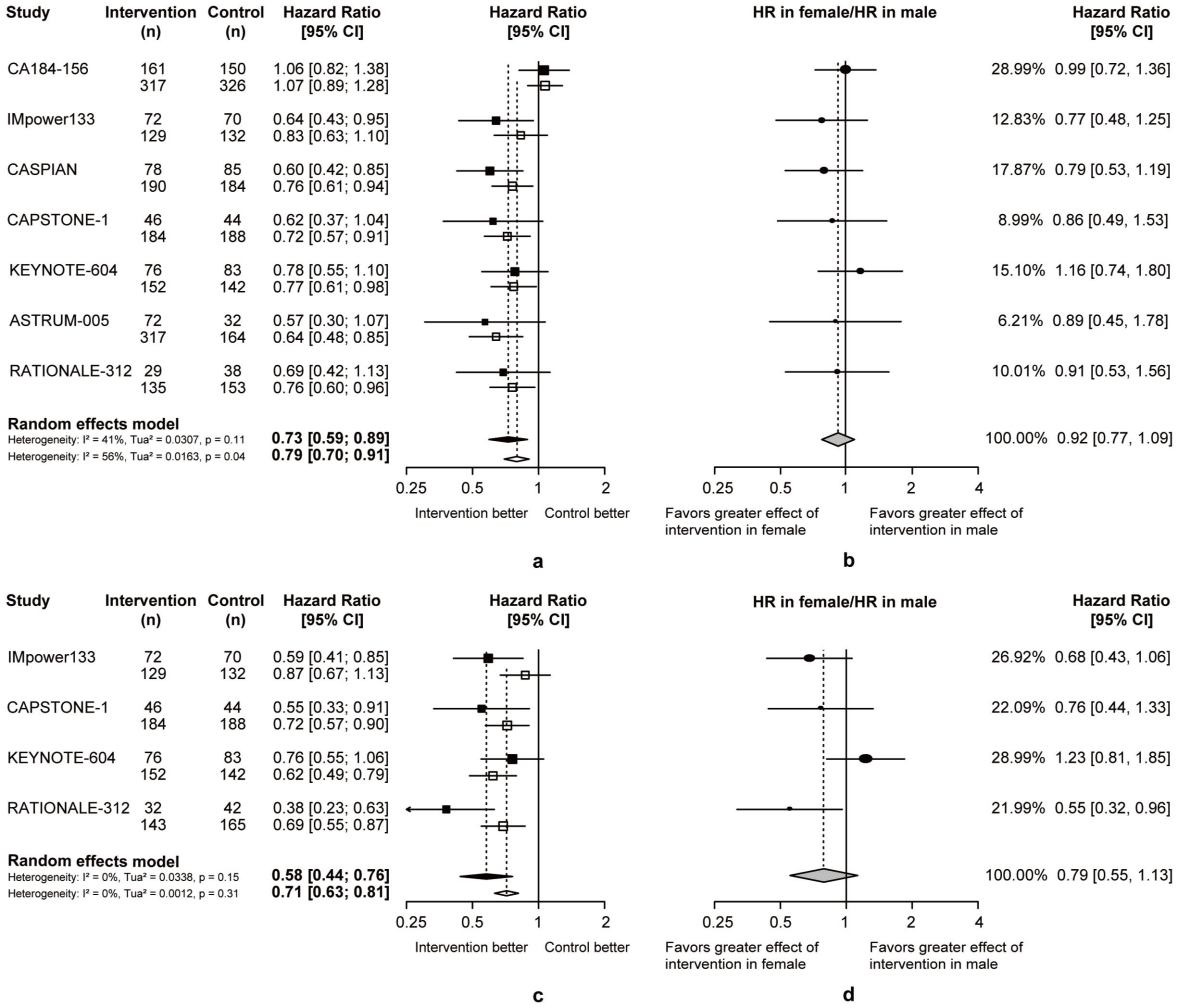
Heterogeneity of efficacy between gender subgroup. **(A)** The OS-HRs of the intervention and control groups are compared in gender subgroup. **(B)** The PFS-HRs of the intervention and control groups are compared in gender subgroup. Squares indicate study-specific hazard ratios. Values less than 1 indicate intervention is better than control. Size of the square is proportional to the precision of the estimate. Horizontal lines indicate the 95% CI. Diamonds indicate the meta-analytic pooled HRs, calculated separately in women and men, with their corresponding 95% CIs. The dashed line represents the specific combined risk ratio of gender subgroup, and the solid line represents a risk ratio of 1, which is the null hypothesis value. **(C)** The pooled ratio of OS-HRs reported in gender subgroup. **(D)** The pooled ratio of PFS-HRs reported in gender subgroup. Each filled circle indicates the study-specific ratio of HRs. Values less than 1 indicate that the effect of the intervention compared with control is greater for women than men. Size of the circle is proportional to the precision of the estimate. Horizontal lines indicate the 95% CI. The diamond indicates the meta-analytic pooled ratio of HRs, with its corresponding 95% CI. The solid line represents a risk ratio of 1, which is the null hypothesis value.

Women and men benefited more from chemoimmunotherapy than chemotherapy in ES-SCLC. (women: pooled OS-HR = 0.82, 95% CI = 0.65 to 1.05, pooled PFS-HR = 0.70, 95% CI = 0.54 to 0.90, men: pooled OS-HR = 0.64, 95% CI = 0.55 to 0.74, pooled PFS-HR = 0.66, 95% CI = 0.56 to 0.79; **Figures 3A, B**). The pooled ratio of OS-HRs in women versus men reported in each trial was 0.92 (95% CI = 0.77 to 1.09; **Figure 3C**), and the pooled ratio of PFS-HRs was 0.79 (95% CI = 0.55 to 1.13; **Figure 3D**). It suggested that women tend to benefit more from first-line chemoimmunotherapy in ES-SCLC.

**Figure 4:**
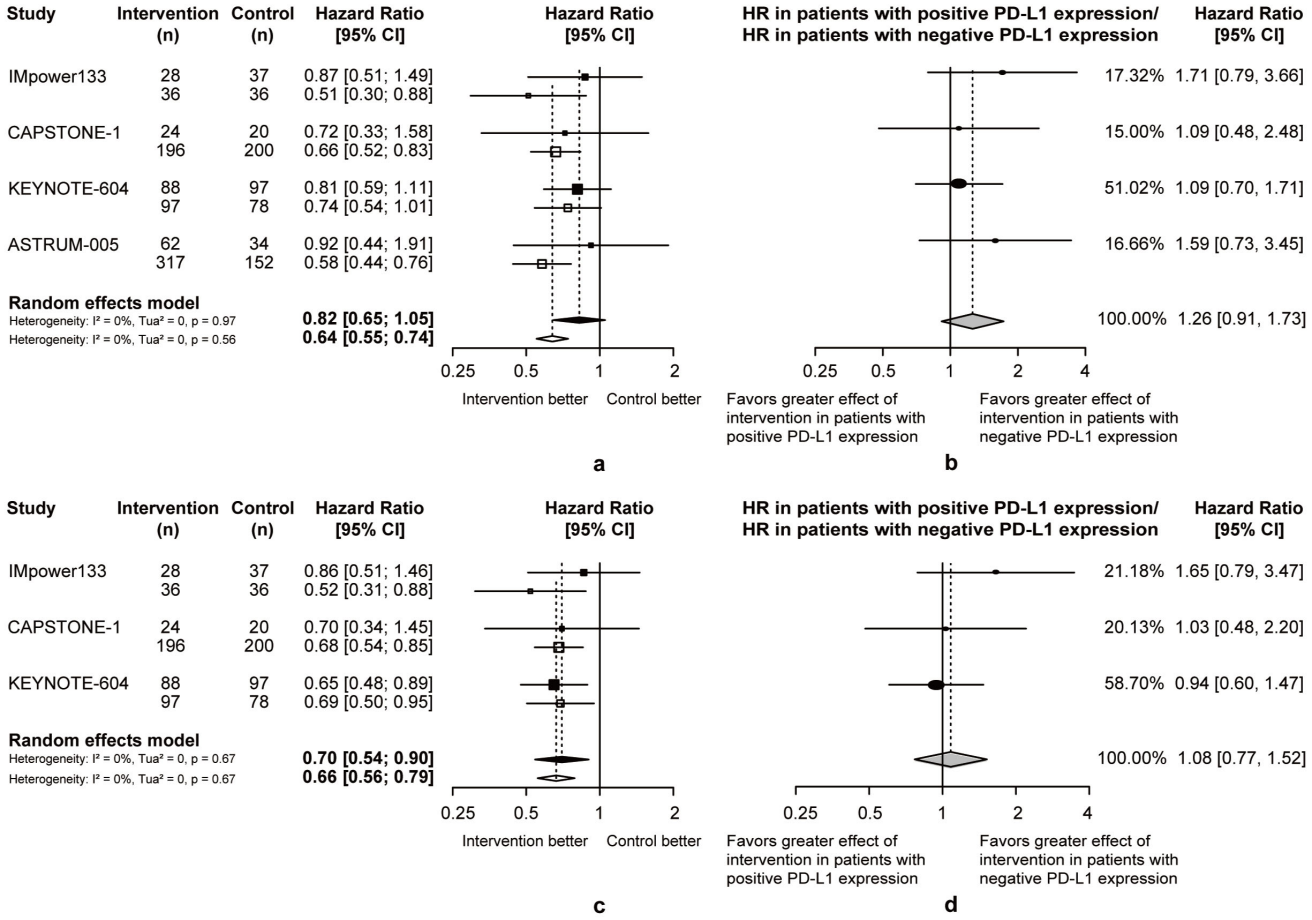
Heterogeneity of efficacy between PD-L1 expression level subgroup. **(A)** The OS-HRs of the intervention and control groups are compared in PD-L1 expression level subgroup. **(B)** The PFS-HRs of the intervention and control groups are compared in PD-L1 expression level subgroup. Squares indicate study-specific hazard ratios. Values less than 1 indicate intervention is better than control. Size of the square is proportional to the precision of the estimate. Horizontal lines indicate the 95% CI. Diamonds indicate the meta-analytic pooled HRs, calculated separately in patients with positive PD-L1 expression and patients with negative PD-L1 expression, with their corresponding 95% CIs. The dashed line represents the specific combined risk ratio of PD-L1 expression level subgroup, and the solid line represents a risk ratio of 1, which is the null hypothesis value. **(C)** The pooled ratio of OS-HRs reported in PD-L1 expression level subgroup. **(D)** The pooled ratio of PFS-HRs reported in PD-L1 expression level subgroup. Each filled circle indicates the study-specific ratio of HRs. Values greater than 1 indicate that the effect of the intervention compared with control is greater for patients with negative PD-L1 expression than patients with positive PD-L1 expression. Size of the circle is proportional to the precision of the estimate. Horizontal lines indicate the 95% CI. The diamond indicates the meta-analytic pooled ratio of HRs, with its corresponding 95% CI. The solid line represents a risk ratio of 1, which is the null hypothesis value.

Compared to chemotherapy, both patients with positive or negative PD-L1 expression benefit more from OS (PD-L1+: pooled OS-HR = 0.82, 95% CI = 0.65 to 1.05, PD-L1-: pooled OS-HR = 0.64, 95% CI = 0.55 to 0.74; **Figure 4A**) and PFS (PD-L1+: pooled PFS-HR = 0.70, 95% CI = 0.54 to 0.90, PD-L1-: pooled PFS-HR = 0.66, 95% CI = 0.56 to 0.79; **Figure 4B**) in chemoimmunotherapy. Respectively, the pooled ratios of OS-HRs and PFS-HRs reported in patients with positive versus negative PD-L1 expression were 1.26 (95% CI = 0.91 to 1.73; **Figure 4C**) and 1.08 (95% CI = 0.77 to 1.52; **Figure 4D**), and this heterogeneity indicated that ES-SCLC patients with negative PD-L1 expression may be more suitable candidates for chemoimmunotherapy.

**Figure 5:**
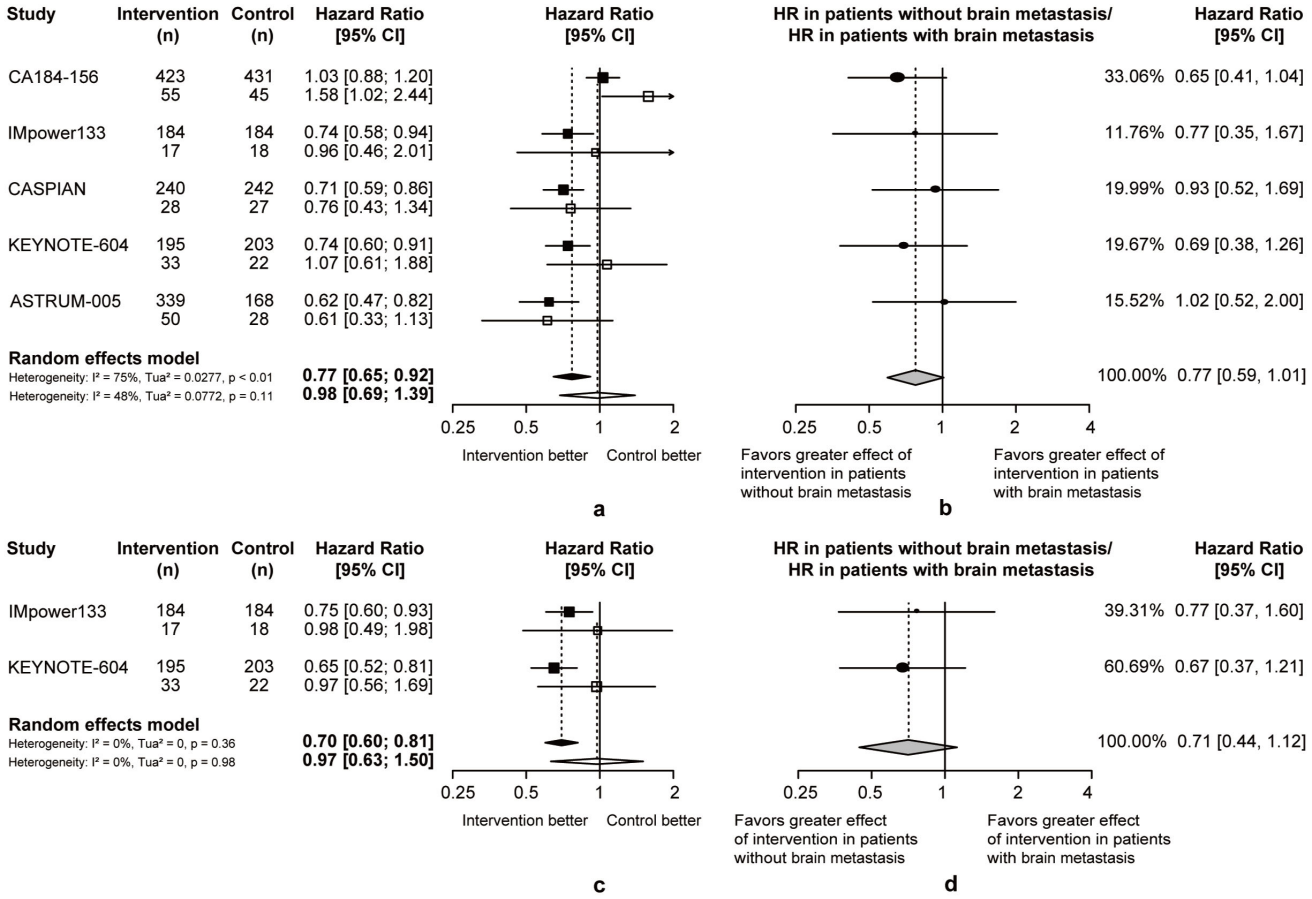
Heterogeneity of efficacy between brain metastases subgroup. **(A)** The OS-HRs of the intervention and control groups are compared in brain metastases subgroup. **(B)** The PFS-HRs of the intervention and control groups are compared in brain metastases subgroup. Squares indicate study-specific hazard ratios. Values less than 1 indicate intervention is better than control. Size of the square is proportional to the precision of the estimate. Horizontal lines indicate the 95% CI. Diamonds indicate the meta-analytic pooled HRs, calculated separately in patients without brain metastasis and patients with brain metastasis, with their corresponding 95% CIs. The dashed line represents the specific combined risk ratio of brain metastases subgroup, and the solid line represents a risk ratio of 1, which is the null hypothesis value. **(C)** The pooled ratio of OS-HRs reported in brain metastases subgroup. **(D)** The pooled ratio of PFS-HRs reported in brain metastases subgroup. Each filled circle indicates the study-specific ratio of HRs. Values less than 1 indicate that the effect of the intervention compared with control is greater for patients without brain metastasis than patients with brain metastasis. Size of the circle is proportional to the precision of the estimate. Horizontal lines indicate the 95% CI. The diamond indicates the meta-analytic pooled ratio of HRs, with its corresponding 95% CI. The solid line represents a risk ratio of 1, which is the null hypothesis value.

In patients with or without brain metastasis, chemoimmunotherapy demonstrated superior efficacy than chemotherapy. Considering the heterogeneity between two groups, the pooled ratios of OS-HRs and PFS-HRs in patients without or with brain metastasis were calculated (pooled ratio of OS-HRs = 0.77, 95% CI = 0.59 to 1.01; pooled ratio of PFS-HRs = 0.71, 95% CI = 0.44 to 1.12; **Figure 5**). This heterogeneity indicated that ES-SCLC patients without brain metastases may achieve better survival outcomes from chemoimmunotherapy.

We performed several similar analyses to assess therapeutic heterogeneity on other clinicopathological characteristics (**Supplementary Figures 1**-**7**). Eventually, we concluded that non-smokers, Asians, patients older than 65 years, patients without liver metastasis, patients with LDH below upper limit of normal (ULN) or using etoposide-cisplatin may have longer OS from chemoimmunotherapy with no statistically significant differences.

### 3.4 Heterogeneity between subgroups of different types of chemoimmunotherapy as the first-line treatment of ES-SCLC patients

Therapeutic heterogeneity between subgroups was analyzed for different treatment regimens, including cytotoxic T lymphocyte-associated antigen-4 (CTLA-4) inhibitors plus chemotherapy, PD-L1 inhibitors plus chemotherapy, and PD-1 inhibitors plus chemotherapy, to identify the dominant population for each regimen (**Table 3**). Statistically significant pooled ratios of PFS-HRs (0.58, 95% CI = 0.34 to 0.99; **Table 3**) indicated that smokers were more suitable for PD-1 inhibitors plus chemotherapy than non-smokers. No statistically significant differences were observed in other subgroups.

**Table 3:** Subgroup analysis of overall survival and progression-free survival among different types of chemoimmunotherapy in this meta-analysis.

	HR (95% CI)									
	Sex	Age	PD-L1 expression level	Brain mts	Liver mts	ECOG PS	LDH level	Race	Smoking status	Platinum salt
OS										
CTLA-4Inhibitors	0.99 (0.72-1.36)	1.06 (0.76-1.47)	NA	0.65 (0.41-1.04)	NA	0.77 (0.56-1.07)	1.02 (0.76-1.37)	NA	0.94 (0.68-1.28)	0.82 (0.59-1.12)
PD-L1Inhibitors	0.80 (0.61-1.05)	0.91 (0.64-1.29)	1.39 (0.79-2.42)	0.87 (0.54-1.40)	0.80 (0.63-1.02)	1.01 (0.77-1.33)	1.41 (0.90-2.19)	0.88 (0.53-1.46)	0.90 (0.58-1.38)	0.88 (0.58-1.34)
PD-1Inhibitors	1.02 (0.75-1.38)	0.89 (0.69-1.15)	1.20 (0.82-1.76)	0.82 (0.52-1.28)	1.05 (0.78-1.41)	1.13 (0.82-1.56)	1.10 (0.82-1.47)	1.28 (0.88-1.88)	0.93 (0.69-1.26)	0.97 (0.70-1.35)
PFS										
PD-L1Inhibitors	0.71 (0.50-1.01)	0.94 (0.69-1.29)	1.31 (0.77-2.23)	0.77 (0.37-1.60)	0.88 (0.65-1.21)	0.94 (0.65-1.34)	0.91 (0.60-1.38)	NA	0.58 (0.34-0.99)	NA
PD-1Inhibitors	0.84 (0.38-1.84)	1.11 (0.83-1.48)	0.94 (0.60-1.47)	0.67 (0.37-1.21)	0.78 (0.58-1.05)	1.06 (0.75-1.52)	1.10 (0.82-1.46)	NA	0.91 (0.65-1.28)	NA

OS, overall survival; PFS, progression-free survival; HR, hazard ratio; CI, confidence interval; CTLA-4, cytotoxic T lymphocyte-associated antigen-4; PD-L1, programmed death-ligand 1; PD-1, programmed death-1; ECOG PS, Eastern Cooperative Oncology Group Performance Sstatus; LDH, lactate dehydrogenase; mts, metastases; NA, not available.

### 3.5 Sensitivity analysis and publication bias

We performed sensitivity analyses on subgroups by gender, PD-L1 expression level, brain metastasis, LDH level, the type of platinum salt and race **(Supplementary Figure 8**). Statistically significant pooled ratios of OS-HRs (0.74, 95%CI = 0.55 to 0.98) in brain metastases subgroup indicated that ES-SCLC patients without brain metastases were the dominant population for first-line chemoimmunotherapy. The pooled ratios of PFS-HRs in gender subgroup (0.66, 95% CI = 0.49-0.89) was statistically significant, indicating that women were more suitable for first-line chemoimmunotherapy. The funnel plot for publication bias was shown in the **Supplementary Figure 10**, and no significant publication bias was observed.

## 4 Discussion

This meta-analysis, which included 9 RCTs, demonstrated that first-line chemoimmunotherapy was more effective than chemotherapy in ES-SCLC patients. Not coincidentally, this result was consistent with the conclusion of a meta-analysis of 6 RCTs published in 2021 (28). Gristina et al. found that specific patient clinical characteristics (such as ECOG PS of 1, the use of cisplatin and the absence of brain metastases) seemed to be associated with a survival gain using chemoimmunotherapy in ES-SCLC patients, and patients both with and without liver metastases receiving chemoimmunotherapy may have better survival outcomes (28). Based on this study, we used the deft approach to directly compare the potential therapeutic heterogeneity between subgroups, which could further assist ES-SCLC patients to choose personalized treatment. By analyzing the therapeutic heterogeneity between subgroups, we concluded that women or non-smokers or Asians or patients over 65 years old or with negative PD-L1 expression or with LDH ≤ ULN or without brain metastasis or without liver metastasis or using etoposide-cisplatin may achieve longer OS from first-line chemoimmunotherapy. Among them, specific patient clinical characteristics tended to obtain longer OS and PFS, including women or patients with negative PD-L1 expression or with LDH ≤ ULN or without brain metastasis. Notably, the OS prolongation trends of patients with negative PD-L1 expression or with LDH ≤ ULN in all RCTs were completely consistent, and the PFS prolongation trends of patients without brain or liver metastasis were completely consistent.

Our analysis of the difference in the efficacy of chemoimmunotherapy between women and men was similar to a previous meta-analysis which showed that women with advanced lung cancer achieved more statistically significant survival improvement from PD-1/PD-L1 inhibitors plus chemotherapy than men (29). Except for KEYNOTE-604 trial, other trials were observed a consistent trend that women receiving chemoimmunotherapy may have better survival outcomes than men. After sensitivity analysis, the PFS improvement of women undergoing chemoimmunotherapy was observed to be statistically significant. Given the complexity of sex-dimorphism of immune system function and responses, women may benefit more than men from different immunotherapy strategies (30). The possible mechanisms underlying this gender heterogeneity include: First, the X chromosome contained immune-related genes that can escape X chromosome inactivation. Second, sex hormone-induced signaling pathways could be regulated by sex chromosome-linked genes. Moreover, PD-1 expression and function could be regulated by estrogen, and PD-L1 was expressed in an estrogen-dependent and sex-dependent manner (31–34). A study using a cRaf transgenic disease model assessed commonalities in sex-specific NSCLC gene regulations between mice and humans, and confirmed the role of estrogen receptor α in affecting immune cells in the tumor microenvironment and regulating tumor growth genes (35). Up to now, several studies have shown that gender heterogeneity should be considered in chemoimmunotherapy for lung cancer patients.

We analyzed the therapeutic heterogeneity of PD-L1 expression level subgroup, and concluded that patients with negative PD-L1 expression may have better survival outcomes. Detection of PD-L1 expression level can guide the use of PD-1/PD-L1 inhibitors and assist to screen potential candidates of immunotherapy. High PD-L1 expression will reduce the immunity of patients, especially in solid tumors, which may seriously affect the survival benefit of patients (36). A phase III RCT has demonstrated that advanced NSCLC patients with high PD-L1 expression and high immune infiltration were the dominant population for chemoimmunotherapy (37). However, a recent study suggested that the predictive value of PD-L1 expression level was not significantly heterogeneity between squamous cell carcinoma and adenocarcinoma patients receiving ICIs plus chemotherapy (38). The aforementioned results were not consistent with our study, which focused on ES-SCLC. Except for KEYNOTE-604 trial, results were consistently observed that patients with negative PD-L1 expression may have longer OS and PFS from chemoimmunotherapy. As a biomarker for ICIs treatment, PD-L1 expression level has been used as an auxiliary diagnosis in selecting immunotherapy options for NSCLC patients (11), but it is not suitable to predict the efficacy of immunotherapy in SCLC. Possible reasons for this difference were as follows: First, the heterogeneity of tumor immune microenvironment in SCLC and NSCLC affects the clinical efficacy of chemoimmunotherapy (39). Second, the expression level of PD-L1 in SCLC was generally lower than that in NSCLC. In Checkmate-032 trial (40), PD-L1 expression was observed to be greater than 1% in only 17% of ES-SCLC patients, and greater than 5% in only 5% of ES-SCLC patients. The sample size of patients with positive PD-L1 expression accounted for only 26% in our meta-analysis, potentially limiting the assessment of therapeutic heterogeneity regarding PD-L1 expression level. Moreover, there were various evaluation approaches and detection methods for PD-L1 expression. For example, PD-L1 expression of tissues in SCLC could not be reflected by fine needle aspiration specimens (41). More studies are needed to explore the feasibility of using PD-L1 expression level as a biomarker in ES-SCLC patients receiving chemoimmunotherapy.

In the heterogeneity analysis of brain metastasis subgroup, patients without brain metastasis may respond better to first-line chemoimmunotherapy than those with brain metastasis. A study of PD-1 inhibitors plus chemotherapy as first-line treatment for advanced nonsquamous NSCLC with brain metastases showed favorable intracranial anti-tumor activity and tolerability of this regimen. For patients with negative PD-L1 expression, this regimen also demonstrated efficacy which provided strong evidence to support the application of chemoimmunotherapy in patients with brain metastasis (42). The study of Rudin et al. similarly found that ES-SCLC patients without brain metastases were the dominant group of first-line chemoimmunotherapy (1). Notably, except for ASTRUM-005 trial, there was a trend that patients without brain metastases receiving chemoimmunotherapy have more significant survival benefits. Varied criteria for brain metastases in the included studies may account for this discrepancy. For example, patients with asymptomatic and stable brain metastases were included in ASTRUM-005 trial (13), while patients with lesions confined to the supratentorial region and cerebellum and without central nervous system progression after stereotactic treatment or whole brain radiotherapy were also included in CAPSTONE-1 trial. In our meta-analysis, patients with brain metastases accounted for only 9.14% of all included patients, which may limit the generalizability even statistical significance of the results. Additionally, due to complex tumor microenvironment of brain metastases and different ability of ICIs to penetrate the blood-brain barrier (43), whether chemoimmunotherapy can be used as the first-line treatment for ES-SCLC patients with brain metastases remains to be studied.

Non-smokers, Asians, patients without liver metastasis or with LDH ≤ UNL or using etoposide-cisplatin were observed to tend to achieve longer OS from chemoimmunotherapy, although these findings did not reach statistical significance. However, the OS and PFS benefits of patients in the age and ECOG PS subgroups were inconsistent after receiving chemoimmunotherapy. Different from our results, a meta-analysis showed that smokers receiving chemoimmunotherapy had a better therapeutic effect than non-smokers in metastatic NSCLC (44). Recently, a study revealed the distinct immune microenvironment of lung adenocarcinoma in non-smokers and smokers, further explaining the poor response of non-smokers to PD-1/PD-L1 inhibitors (45). Similarly, the distinct immune microenvironment of SCLC and NSCLC may lead to different efficacy of immunotherapy for smokers. The reliable efficacy and safety of first-line chemoimmunotherapy in ES-SCLC patients have been confirmed by several real-world researches (RWR) of different regions (46–50), but some studies on the therapeutic heterogeneity of different ethnicities lacked sufficient evidence. A meta-analysis in NSCLC and SCLC patients with liver metastasis indicated that the presence of liver metastases did not significantly affect the OS benefit of ICIs in NSCLC patients, while a small amount of data showed that liver metastasis inhibited OS benefit in SCLC patients (51). By developing a two-site tumor system model, a study found that liver metastasis can inhibit systemic anti-tumor immune response, and proposed that CTLA-4 inhibitors or enhancer of zeste homolog 2 (EZH2) inhibitors combined with PD-1 inhibitors can restore systemic anti-tumor immune activity (52). Therefore, PD-1 inhibitors combined with targeted drugs may be required for patients with liver metastasis. The study of R. Zeng et al. found that OS benefit of PD-L1/PD-1 inhibitors plus chemotherapy in ES-SCLC patients with LDH ≤ ULN was superior to those with LDH > ULN (53). High level of LDH expression has been reported to promote epithelial to mesenchymal transition (54), angiogenesis, cellular invasion and migration, which is associated with a poor prognosis in patients with various solid tumors (55). According to the guidelines, etoposide-carboplatin is selected in more cases (56). Furthermore, a study has shown that the survival advantage associated with cisplatin was not superior to that of carboplatin in single chemotherapy regimen of ES-SCLC, and less toxic carboplatin-etoposide plus chemotherapy regimen may be better (57). In future clinical studies, more patients using etoposide-cisplatin should be considered to provide better treatment options for ES-SCLC patients. A study of chemoimmunotherapy for advanced NSCLC showed that age was negatively correlated with survival in patients receiving ICIs combined with or without chemotherapy, indicating that the differential use of chemoimmunotherapy across age groups was unlikely to account for age-related survival differences (58). A previous meta-analysis also analyzed the impact of age on the efficacy of chemoimmunotherapy in lung cancer patients, and no statistically significant effect was observed (29). For most RCTs, the majority of patients included were ECOG PS 0 or 1, and patients with ECOG PS ≥ 2 were usually excluded, leading to unclear efficacy of chemoimmunotherapy in these patients. A meta-analysis of 19 studies containing 3600 NSCLC patients showed that the efficacy of chemoimmunotherapy in patients with ECOG PS ≥ 2 was comparable to that of chemotherapy (59). Regardless of ECOG PS, ICIs have been approved and routinely administered, so whether ECOG PS is a predictor of chemoimmunotherapy efficacy remains to be confirmed.

One advantage of the analysis is that all the data were derived from large RCTs with similar trial designs and enrolled populations. Additionally, our meta-analysis is the latest and most detailed assessment of heterogeneity between subgroups of first-line chemoimmunotherapy for ES-SCLC patients, including therapeutic heterogeneity among clinicopathological characteristics and subgroup analyses of different chemoimmunotherapy regimens. Notably, patients with negative PD-L1 expression may have better survival outcomes than those with positive PD-L1 expression. Although this result did not reach a statistically significant level, it provided guidance for the treatment of ES-SCLC patients and underscores the need for further clinical and basic research to explore the significance of PD-L1 expression in ES-SCLC.

Nevertheless, there are several limitations in our meta-analysis. First, this meta-analysis is based on published clinical trial data and lacks individual patient-level data, which hinders more in-depth analysis and may lead to potential publication bias. Second, although all included trials are RCTs, the imbalance of baseline characteristics (selected patient population, sample size, low incidence, different treatment of brain metastases, etc.) of included trials should be considered. Third, these results should always be interpreted with caution since the included trials are subject to updates and several ongoing trials are not included.

In conclusion, we suggested that chemoimmunotherapy can significantly prolong OS and PFS in ES-SCLC patients compared with chemotherapy. By analyzing the therapeutic heterogeneity between subgroups, we concluded that women or patients with negative PD-L1 expression or with LDH ≤ ULN or without brain metastasis tend to benefit more from first-line chemoimmunotherapy in ES-SCLC. Additionally, patients with negative PD-L1 expression or LDH ≤ ULN have consistent trend toward prolonged OS, and patients without brain metastasis or liver metastasis have consistent trend toward prolonged PFS. In aggregate, the findings of this meta-analysis could assist in achievement of personalized treatment by screening out more suitable candidates for chemoimmunotherapy, as well as the design and interpretation of future trials on therapeutic heterogeneity in ES-SCLC patients.

## Data Availability

The original contributions presented in the study are included in the article/**Supplementary Material**. Further inquiries can be directed to the corresponding authors.
