# Supplementary material for: Heterogeneity between subgroups of first-line chemoimmunotherapy for extensive-stage small cell lung cancer patients: a meta-analysis and systematic review
Source: Front Oncol. 2024 Oct 18;14:1334957. doi: 10.3389/fonc.2024.1334957 (PMC11527596; doi:10.3389/fonc.2024.1334957)
Supplement: Supplementary file 1 [file DataSheet1.pdf]

Supplementary Material

1 Supplementary Tables and Figures

1.1 Supplementary Tables

Supplementary Table 1. Search strategy

**PubMed**	
#1	(((((("Small Cell Lung Carcinoma"[Mesh]) OR ("Small Cell Lung Cancer"[Title/Abstract])) OR (SCLC[Title/Abstract])) AND (((((((("Neoplasm Metastasis"[MeSH]) OR ("Neoplasm Metastases"[Title/Abstract])) OR (metastase[Title/Abstract])) OR (metastases[Title/Abstract])) OR (metastatic[Title/Abstract])) OR (extensive[Title/Abstract])) OR (stage IV[Title/Abstract])) OR (stage 4[Title/Abstract])) OR (stage four[Title/Abstract])) OR ("Extensive stage"[Title/Abstract]))) OR ("extensive stage small cell lung cancer"[Title/Abstract])) OR (ES-SCLC[Title/Abstract])
#2	((((("Combined Modality Therapy"[Mesh]) OR ("Multimodal Treatment"[Title/Abstract])) OR ("Multimodal Treatments"[Title/Abstract])) OR ("Combined Modality Therapies"[Title/Abstract])) OR ("immunotherapy plus chemotherapy"[Title/Abstract])) OR (Chemoimmunotherapy[Title/Abstract])
#3	((((((((((((Immunotherapy[Mesh]) OR (immunotherapies[Title/Abstract])) OR (((((((((((((((((((((((((((("Immune Checkpoint Inhibitors"[Mesh]) OR ("Immune Checkpoint Inhibitor")) OR ("Immune Checkpoint Blockers")) OR ("Immune Checkpoint Blockade")) OR ("Immune Checkpoint Inhibition")) OR ("PD-L1 Inhibitors")) OR ("PD L1 Inhibitors")) OR ("PD-L1 Inhibitor")) OR ("PD L1 Inhibitor")) OR ("Programmed Death-Ligand 1 Inhibitors")) OR ("Programmed Death Ligand 1 Inhibitors")) OR ("PD-1-PD-L1 Blockade")) OR ("PD 1 PD L1 Blockade")) OR ("CTLA-4 Inhibitors")) OR ("CTLA 4 Inhibitors")) OR ("CTLA-4 Inhibitor")) OR ("CTLA 4 Inhibitor")) OR ("Cytotoxic T-Lymphocyte-Associated Protein 4 Inhibitors")) OR ("Cytotoxic T Lymphocyte Associated Protein 4 Inhibitors")) OR ("Cytotoxic T-Lymphocyte-Associated Protein 4 Inhibitor")) OR ("Cytotoxic T Lymphocyte Associated Protein 4 Inhibitor")) OR ("PD-1 Inhibitors")) OR ("PD 1 Inhibitors")) OR ("PD-1 Inhibitor")) OR ("PD 1 Inhibitor")) OR ("Programmed Cell Death Protein 1 Inhibitor")) OR ("Programmed Cell Death Protein 1 Inhibitors")) OR (ICIs)) OR (ICI))) OR<br>((((pembrolizumab[Supplementary Concept]) OR (Pembrolizumab)) OR (Keytruda)) OR (lambrolizumab))) OR<br>(((nivolumab[MeSH Terms]) OR (Nivolumab)) OR (Opdivo))) OR<br>(Serplulimab[Title/Abstract])) OR<br>((cemiplimab[Supplementary Concept]) OR (Cemiplimab))) OR<br>(((atezolizumab[Supplementary Concept]) OR (Atezolizumab)) OR (Tecentriq))) OR<br>(((durvalumab[Supplementary Concept]) OR (Durvalumab)) OR (Imfinzi))) OR<br>(Adebrelimab[Title/Abstract])) OR<br>(((avelumab[Supplementary Concept]) OR (Avelumab)) OR (bavencio))) OR<br>(((ipilimumab[MeSH Terms]) OR (Ipilimumab)) OR (Yervoy))) OR<br>(((tremelimumab[Supplementary Concept]) OR (Tremelimumab)) OR (ticilimumab))) AND<br>((((((((("Drug Therapy"[Mesh]) OR ("Drug Therapies"[Title/Abstract])) OR (Chemotherapy[Title/Abstract])) OR (Chemotherapies[Title/Abstract])) OR (Pharmacotherapy[Title/Abstract])) OR (Pharmacotherapies[Title/Abstract])) OR (Etoposide[Title/Abstract])) OR (Cisplatin[Title/Abstract])) OR (Carboplatin[Title/Abstract])) OR (Paclitaxel[Title/Abstract]))
#4	(((((((randomized controlled trial[Publication Type]) OR (controlled clinical trial[Publication Type])) OR (randomized[Title/Abstract])) OR (placebo[Title/Abstract])) OR (randomly[Title/Abstract])) OR (trial[Title/Abstract])) OR (clinical[Title/Abstract])) OR (groups[Title/Abstract])
#5	#2 OR #3
#6	#1 AND #5 AND #4
	**2130**

**Cochrane**	
#1	MeSH descriptor: [Small Cell Lung Carcinoma] explode all trees
#2	("Small Cell Lung Carcinoma" OR "Small Cell Cancer Of The Lung" OR "Small Cell Lung Cancer" OR SCLC):ti,ab,kw
#3	#1 OR #2
#4	MeSH descriptor: [Neoplasm Metastasis] explode all trees
#5	("Neoplasm Metastases" OR metastase OR metastases OR metastatic OR extensive OR "stage IV" OR "stage 4" OR "stage four" OR "Extensive stage"):ti,ab,kw
#6	#4 OR #5
#7	("extensive stage small cell lung cancer" OR ES-SCLC):ti,ab,kw
#8	#6 AND #3
#9	#7 OR #8
#10	MeSH descriptor: [Combined Modality Therapy] explode all trees

#11	("Combined Modality Therapy" OR "Multimodal Treatment" OR "Multimodal Treatments" OR "Combined Modality Therapies" OR "immunotherapy plus chemotherapy" OR Chemoimmunotherapy):ti,ab,kw
#12	#10 OR #11
#13	MeSH descriptor: [Immunotherapy] explode all trees
#14	(Immunotherapy OR immunotherapies):ti,ab,kw
#15	#13 OR #14
#16	MeSH descriptor: [Immune Checkpoint Inhibitors] explode all trees
#17	("Immune Checkpoint Inhibitors" OR " Immune Checkpoint Inhibitor" OR "Immune Checkpoint Blockers" OR "Immune Checkpoint Blockade" OR "Immune Checkpoint Inhibition" OR ICI OR ICIs):ti,ab,kw
#18	#16 OR #17
#19	MeSH descriptor: [Programmed Cell Death 1 Receptor] explode all trees
#20	("Programmed Cell Death 1 Receptor" OR "Programmed Cell Death 1 Protein" OR "Programmed Cell Death Protein 1" OR "PD-1 Receptor" OR CD279 Antigen)
#21	#19 OR #20
#22	MeSH descriptor: [B7-H1 Antigen] explode all trees
#23	(PD-L1 OR "Programmed Cell Death 1 Ligand 1 Protein" OR "CD274 Antigens" OR "Costimulatory Protein" OR "Immune Costimulatory Protein")
#24	#22 OR #23
#25	MeSH descriptor: [CTLA-4 Antigen] explode all trees
#26	("CTLA-4 Antigen" OR CTLA-4 OR "Cytotoxic T-Lymphocyte Associated Antigen 4" OR "Cytotoxic T-Lymphocyte Antigen 4" OR "CD152 Antigen")
#27	#25 OR #26
#28	(Pembrolizumab OR Keytruda OR Lambrolizumab)
#29	(Nivolumab OR Opdivo)
#30	(Serplulimab)
#31	(Cemiplimab)
#32	(Atezolizumab OR Tecentriq)
#33	(Durvalumab OR Imfinzi)
#34	(Adebrelimab)
#35	(Avelumab OR Bavencio)
#36	(Ipilimumab OR Yervoy)
#37	(Tremelimumab OR Ticilimumab)
#38	#15 OR #18 OR #21 OR #24 OR #27 OR #28 OR #29 OR #30 OR #31 OR #32 OR #33 OR #34 OR #35 OR #36 OR #37
#39	MeSH descriptor: [Drug Therapy] explode all trees
#40	("Drug Therapy" OR "Drug Therapies" OR Chemotherapy OR Chemotherapies OR Pharmacotherapy OR Pharmacotherapies OR Etoposide OR Cisplatin OR Carboplatin OR Paclitaxel):ti,ab,kw
#41	#39 OR #40
#42	#38 AND #41
#43	#12 OR #42
#44	#9 AND #43
	**1581**

**Embase**	
#1	'small cell lung cancer'/exp
#2	'small cell lung carcinoma':ab,kw,ti OR 'small cell cancer of the lung':ab,kw,ti OR 'small cell lung cancer':ab,kw,ti OR 'sclc':ab,kw,ti
#3	#1 OR #2
#4	'metastasis'/exp
#5	metastasis:ab,kw,ti OR metastase:ab,kw,ti OR metastases:ab,kw,ti OR metastatic:ab,kw,ti OR extensive:ab,kw,ti OR 'stage iv':ab,kw,ti OR 'stage 4':ab,kw,ti OR 'stage four':ab,kw,ti OR 'neoplasm metastases':ab,kw,ti OR 'extensive stage':ab,kw,ti
#6	#4 OR #5
#7	#3 AND #6
#8	'extensive stage small cell lung cancer':ab,kw,ti OR 'es sclc':ab,kw,ti
#9	#7 OR #8
#10	'multimodality cancer therapy'/exp
#11	'multimodality cancer therapy':ab,kw,ti OR 'combined modality therapy':ab,kw,ti OR 'multimodal treatment':ab,kw,ti OR 'multimodal treatments':ab,kw,ti OR 'combined modality therapies':ab,kw,ti OR 'immunotherapy plus chemotherapy':ab,kw,ti OR chemoimmunotherapy:ab,kw,ti
#12	#10 OR #11
#13	'immunotherapy'/exp
#14	immunotherapy:ab,kw,ti OR immunotherapies:ab,kw,ti
#15	#13 OR #14

#16	'immune checkpoint inhibitor'/exp
#17	'immune checkpoint inhibitor':ab,kw,ti OR 'immune checkpoint inhibitors':ab,kw,ti OR 'immune checkpoint blockers':ab,kw,ti OR 'immune checkpoint blockade':ab,kw,ti OR 'immune checkpoint inhibition':ab,kw,ti OR ici:ab,kw,ti OR icis:ab,kw,ti
#18	#16 OR #17
#19	'programmed death 1 receptor'/exp OR 'programmed cell death 1 protein' OR 'programmed cell death protein 1' OR 'pd-1 receptor' OR 'cd279 antigen'
#20	'programmed death 1 ligand 1'/exp OR 'pd l1' OR 'programmed cell death 1 ligand 1 protein' OR 'cd274 antigens'/exp OR 'cd274 antigens' OR 'costimulatory protein' OR 'immune costimulatory protein'
#21	'cytotoxic t lymphocyte antigen 4'/exp OR 'ctla-4 antigen'/exp OR 'ctla-4 antigen' OR 'ctla 4'/exp OR 'ctla 4' OR 'cytotoxic t-lymphocyte associated antigen 4'/exp OR 'cytotoxic t-lymphocyte associated antigen 4' OR 'cytotoxic t-lymphocyte antigen 4'/exp OR 'cytotoxic t-lymphocyte antigen 4' OR 'cd152 antigen'/exp OR 'cd152 antigen'
#22	'pembrolizumab'/exp OR keytruda OR lambrolizumab
#23	'nivolumab'/exp OR opdivo
#24	'serplulimab'/exp
#25	'cemiplimab'/exp
#26	'atezolizumab'/exp OR tecentriq
#27	'durvalumab'/exp OR imfinzi
#28	'adebrelimab'/exp
#29	'avelumab'/exp OR bavencio
#30	'ipilimumab'/exp OR yervoy
#31	'ticilimumab'/exp OR tremelimumab
#32	'chemotherapy'/exp
#33	chemotherapy:ab,kw,ti OR 'drug therapy':ab,kw,ti OR 'drug therapies:ab,kw,ti' OR chemotherapies:ab,kw,ti OR pharmacotherapy:ab,kw,ti OR pharmacotherapies:ab,kw,ti OR etoposide:ab,kw,ti OR cisplatin:ab,kw,ti OR carboplatin:ab,kw,ti OR paclitaxel:ab,kw,ti
#34	#32 OR #33
#35	#15 OR #18 OR #19 OR #20 OR #21 OR #22 OR #23 OR #24 OR #25 OR #26 OR #27 OR #28 OR #29 OR #30 OR #31
#36	#34 AND #35
#37	#12 OR #36
#38	'randomized controlled trial'/exp
#39	'controlled clinical trial'/exp
#40	randomized:ab,kw,ti OR placebo:ab,kw,ti OR randomly:ab,kw,ti OR trial:ab,kw,ti OR clinical:ab,kw,ti OR groups:ab,kw,ti
#41	#38 OR #39 OR #40
#42	#9 AND #37 AND #41
	**4329**

Supplementary Table 2. Subgroup analyses of overall survival for each included trial considered in this meta-analysis

Patient characteristics (OS)		CA184-041 HR(95% CI)	CA184-156 HR(95% CI)	IMpower133 HR(95% CI)	CASPIAN HR(95% CI)	CAPSTONE-1 HR(95% CI)	EA5161 HR(95% CI)	KEYNOTE-604 HR(95% CI)	ASTRUM-005 HR(95% CI)
Sex									
	Male	NA	1.07 (0.89-1.28)	0.83 (0.63-1.10)	0.76 (0.62-0.95)	0.72 (0.57-0.92)	NA	0.77 (0.61-0.98)	0.64 (0.48-0.84)
	Female	NA	1.06 (0.81-1.37)	0.64 (0.43-0.94)	0.60 (0.42-0.84)	0.62 (0.37-1.05)	NA	0.78 (0.55-1.10)	0.57 (0.30-1.06)
Age									
	<65	NA	1.08 (0.90-1.31)	0.94 (0.68-1.28)	0.68 (0.54-0.87)	0.71 (0.54-0.93)	NA	0.88 (0.66-1.17)	0.62 (0.45-0.86)
	≥65	NA	1.14 (0.87-1.49)	0.59 (0.42-0.82)	0.78 (0.59-1.04)	0.70 (0.48-1.00)	NA	0.70 (0.53-0.91)	0.60 (0.40-0.89)
Race									
	Asian	NA	1.16 (0.82-1.64)	NA	0.81 (0.50-1.28)	NA	NA	0.61 (0.38-0.96)	0.58 (0.43-0.79)
	Non-Asian	NA	NA	NA	0.71 (0.58-0.87)	NA	NA	0.82 (0.66-1.02)	0.70 (0.43-1.13)
ECOG PS	0	NA	1.28 (0.98-1.69)	0.73 (0.48-1.10)	0.70 (0.51-0.95)	0.83 (0.46-1.52)	NA	0.74 (0.50-1.10)	0.44 (0.23-0.84)
	1	NA	0.99 (0.83-1.18)	0.78 (0.60-1.03)	0.73 (0.58-0.92)	0.69 (0.55-0.87)	NA	0.80 (0.64-1.00)	0.65 (0.49-0.86)
Platinum salt	Carbo-platin	NA	1.14 (0.96-1.37)	0.76 (0.60-0.95)	0.74 (0.60-0.91)	NA	NA	0.80 (0.63-1.01)	NA
	Cis-platin	NA	0.93 (0.71-1.21)	NA	0.65 (0.45-0.94)	NA	NA	0.71 (0.49-1.02)	NA
Brain mts	Yes	NA	1.58 (1.02-2.44)	0.96 (0.46-2.01)	0.76 (0.43-1.33)	NA	NA	1.07 (0.61-1.88)	0.61 (0.33-1.13)
	No	NA	1.03 (0.88-1.20)	0.74 (0.58-0.94)	0.71 (0.59-0.86)	0.68 (0.55-0.85)	NA	0.74 (0.60- 0.91)	0.62 (0.47-0.82)
Liver mts	Yes	NA	NA	0.75 (0.52-1.07)	0.87 (0.66-1.16)	0.92 (0.65-1.31)	NA	0.77 (0.57-1.03)	NA
	No	NA	NA	0.76 (0.56-1.01)	0.68 (0.53-0.88)	0.61 (0.46-0.81)	NA	0.75 (0.58-0.98)	NA
Smoking status	Smoker	NA	1.09 (0.89-1.32)	NA	0.71 (0.59-0.86)	0.75 (0.59-0.95)	NA	0.81 (0.63-1.04)	0.61 (0.36-1.02)
	Non-Smoker	NA	1.02 (0.80-1.30)	NA	0.82 (0.41-1.69)	0.59 (0.37-0.95)	NA	0.71 (0.49-1.02)	0.75 (0.42-1.33)
LDH									
	≤ULN	NA	1.08 (0.87-1.34)	NA	NA	0.59 (0.42-0.82)	NA	0.73 (0.53-0.99)	NA
	>ULN	NA	1.10 (0.90-1.35)	NA	NA	0.83 (0.62-1.11)	NA	0.79 (0.61-1.02)	NA
PD-L1 ex-pression	<1%	NA	NA	0.51 (0.30-0.89)	NA	0.66 (0.52-0.83)	NA	0.74 (0.54-1.01)	0.58 (0.44-0.76)
	≥1%	NA	NA	0.87 (0.51-1.49)	NA	0.72 (0.33-1.59)	NA	0.81 (0.59-1.11)	0.92 (0.44-1.89)

OS, overall survival; HR, hazard ratio; CI, confidence interval; PD-L1, programmed death-ligand 1; ECOG, Eastern Cooperative Oncology Group; PS, performance status; mts, metastases; LDH, lactate dehydrogenase; ULN, upper limit of normal; NA, not available.

Supplementary Table 3. Subgroup analyses of progression-free survival for each included trial considered in this meta-analysis

Patient characteristics (PFS)		CA184-041 HR(95% CI)	CA184-156 HR(95% CI)	IMpower133 HR(95% CI)	CASPIAN HR(95% CI)	CAPSTONE-1 HR(95% CI)	EA5161 HR(95% CI)	KEYNOTE-604 HR(95% CI)	ASTRUM-005 HR(95% CI)
Sex									
	Male	NA	NA	0.87 (0.67-1.13)	NA	0.72 (0.57-0.90)	NA	0.62 (0.49-0.80)	NA
	Female	NA	NA	0.59 (0.41-0.85)	NA	0.55 (0.33-0.90)	NA	0.76 (0.55-1.06)	NA
Age									
	<65	NA	NA	0.76 (0.57-1.10)	NA	0.70 (0.54-0.91)	NA	0.69 (0.52-0.92)	NA
	≥65	NA	NA	0.76 (0.56-1.03)	NA	0.62 (0.43-0.89)	NA	0.68 (0.52-0.89)	NA
Race									
	Asian	NA	NA	0.84 (0.59-1.20)	NA	0.62 (0.35-1.10)	NA	0.62 (0.42-0.92)	NA
	Non-Asian	NA	NA	0.72 (0.55-0.94)	NA	0.69 (0.56-0.87)	NA	0.70 (0.56-0.88)	NA
ECOG PS	0	NA	NA	0.98 (0.49-2.00)	NA	NA	NA	0.97 (0.56-1.69)	NA
	1	NA	NA	0.75 (0.60-0.93)	NA	0.65 (0.53-0.81)	NA	0.65 (0.52-0.80)	NA
Platinum salt	Carbo-platin	NA	NA	0.80 (0.57-1.13)	NA	0.74 (0.51-1.07)	NA	0.83 (0.62-1.12)	NA
	Cis-platin	NA	NA	0.72 (0.55-0.94)	NA	0.64 (0.50-0.83)	NA	0.60 (0.46-0.77)	NA
Brain mts	Yes	NA	NA	NA	NA	0.76 (0.60-0.96)	NA	0.68 (0.53-0.87)	NA
	No	NA	NA	NA	NA	0.44 (0.27-0.71)	NA	0.72 (0.51-1.02)	NA
Liver mts	Yes	NA	NA	NA	NA	0.70 (0.52-0.95)	NA	0.62 (0.46-0.84)	NA
	No	NA	NA	NA	NA	0.64 (0.48-0.85)	NA	0.73 (0.56-0.94)	NA
Smoking status	Smoker	NA	NA	0.52 (0.31-0.88)	NA	0.68 (0.54-0.85)	NA	0.69 (0.50-0.94)	NA
	Non-Smoker	NA	NA	0.86 (0.51-1.46)	NA	0.70 (0.34-1.45)	NA	0.65 (0.47-0.88)	NA
LDH		NA	NA	0.77 (0.63-0.95)	NA	0.67 (0.54-0.83)	NA	0.70 (0.57-0.85)	NA
	≤ULN	NA	NA	0.87 (0.67-1.13)	NA	0.72 (0.57-0.90)	NA	0.62 (0.49-0.80)	NA
	>ULN	NA	NA	0.59 (0.41-0.85)	NA	0.55 (0.33-0.90)	NA	0.76 (0.55-1.06)	NA
PD-L1 ex-pression	<1%	NA	NA	0.76 (0.57-1.10)	NA	0.70 (0.54-0.91)	NA	0.69 (0.52-0.92)	NA
	≥1%	NA	NA	0.76 (0.56-1.03)	NA	0.62 (0.43-0.89)	NA	0.68 (0.52-0.89)	NA

PFS, progression-free survival; HR, hazard ratio; CI, confidence interval; PD-L1, programmed death-ligand 1; ECOG, Eastern Cooperative Oncology Group; PS, performance status; mts, metastases; LDH, lactate dehydrogenase; ULN, upper limit of normal; NA, not available.

1.2 Supplementary Figures

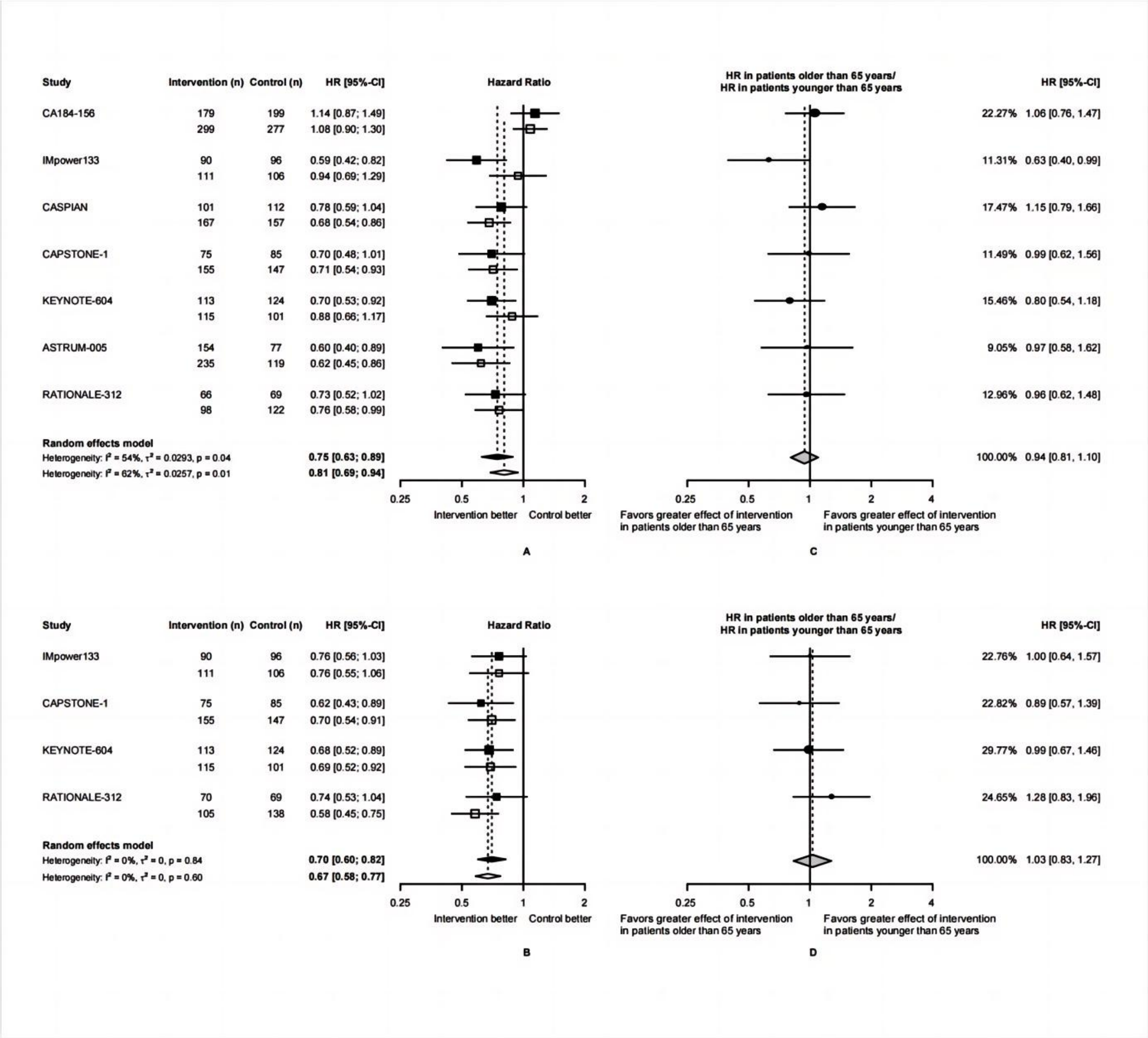

Supplementary Figure 1. Heterogeneity of efficacy between age subgroup. (A) The OS-HRs of the intervention and control groups are compared in age subgroup. (B) The PFS-HRs of the intervention and control groups are compared in age subgroup. Squares indicate study-specific hazard ratios. Values less than 1 indicate intervention is better than control. Size of the square is proportional to the precision of the estimate. Horizontal lines indicate the 95% CI. Diamonds indicate the meta-analytic pooled HRs, calculated separately in patients with age≥65 years and patients with aged<65 years, with their corresponding 95% CIs. The dashed line represents the specific combined risk ratio of age subgroup, and the solid line represents a risk ratio of 1, which is the null hypothesis value. (C) The pooled ratio of OS-HRs reported in age subgroup. (D) The pooled ratio of PFS-HRs reported in age subgroup. Each filled circle indicates the study-specific ratio of HRs. Values less than 1 indicate that the effect of the intervention compared with control is greater for patients with age≥65 years than patients with aged<65 years. Values more than 1 indicate that the effect of the intervention compared with control is greater for patients with age<65 years than patients with aged≥65 years. Size of the circle is proportional to the precision of the estimate. Horizontal lines indicate the 95% CI. The diamond indicates the meta-analytic pooled ratio of HRs, with its corresponding 95% CI. The solid line represents a risk ratio of 1, which is the null hypothesis value.

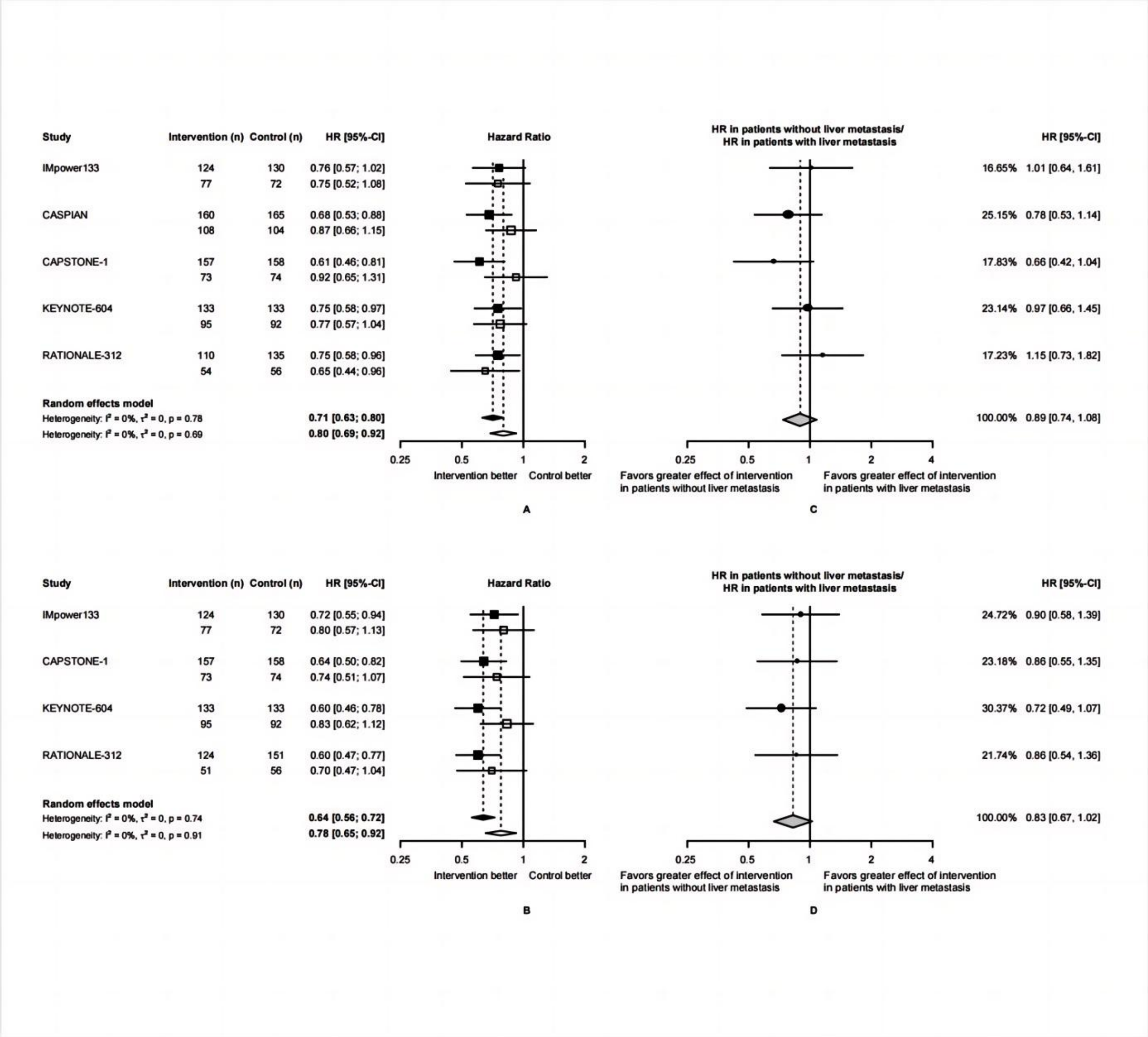

Supplementary Figure 2. Heterogeneity of efficacy between liver metastases subgroup. (A) The OS-HRs of the intervention and control groups are compared in liver metastases subgroup. (B) The PFS-HRs of the intervention and control groups are compared in liver metastases subgroup. Squares indicate study-specific hazard ratios. Values less than 1 indicate intervention is better than control. Size of the square is proportional to the precision of the estimate. Horizontal lines indicate the 95% CI. Diamonds indicate the meta-analytic pooled HRs, calculated separately in patients without liver metastases and patients with liver metastases, with their corresponding 95% CIs. The dashed line represents the specific combined risk ratio of liver metastases subgroup, and the solid line represents a risk ratio of 1, which is the null hypothesis value. (C) The pooled ratio of OS-HRs reported in liver metastases subgroup. (D) The pooled ratio of PFS-HRs reported in liver metastases subgroup. Each filled circle indicates the study-specific ratio of HRs. Values less than 1 indicate that the effect of the intervention compared with control is greater for patients without liver metastases than patients with liver metastases. Size of the circle is proportional to the precision of the estimate. Horizontal lines indicate the 95% CI. The diamond indicates the meta-analytic pooled ratio of HRs, with its corresponding 95% CI. The solid line represents a risk ratio of 1, which is the null hypothesis value.

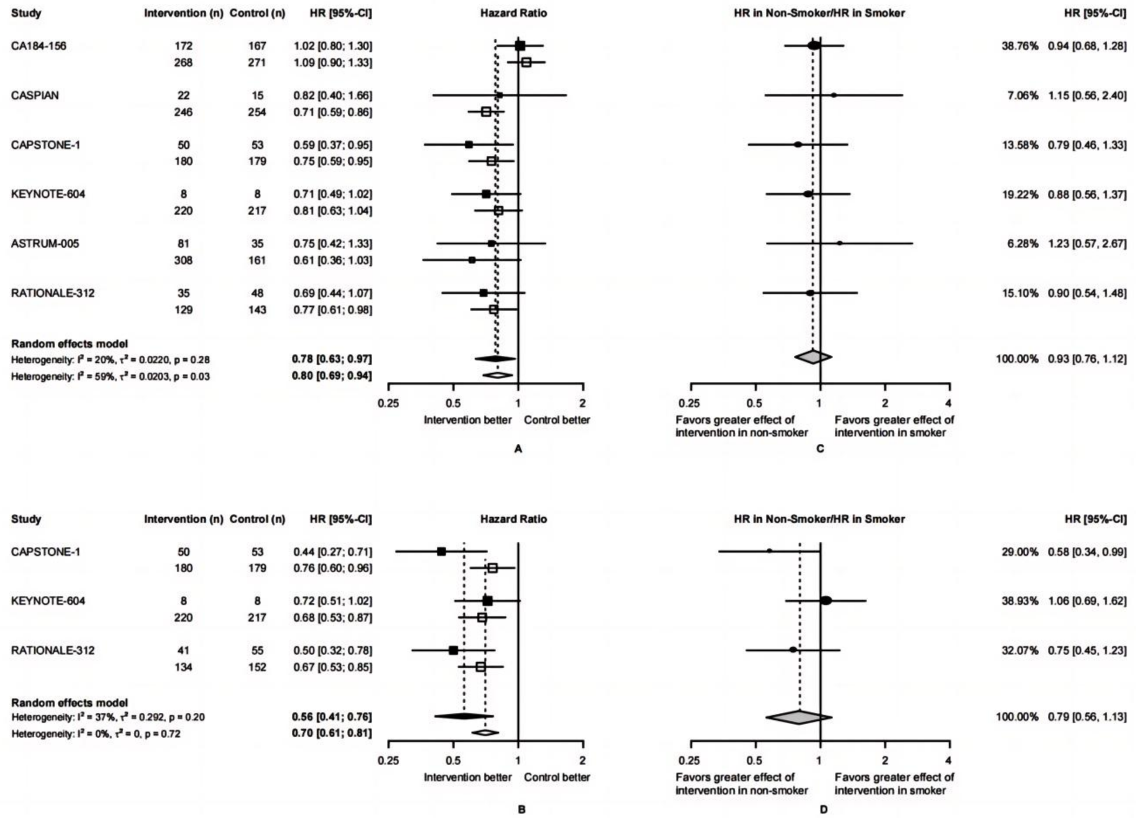

Supplementary Figure 3. Heterogeneity of efficacy between smoking status subgroup. Heterogeneity of efficacy between smoking status subgroup. (A) The OS-HRs of the intervention and control groups are compared in smoking status subgroup. (B) The PFS-HRs of the intervention and control groups are compared in smoking status subgroup. Squares indicate study-specific hazard ratios. Values less than 1 indicate intervention is better than control. Size of the square is proportional to the precision of the estimate. Horizontal lines indicate the 95% CI. Diamonds indicate the meta-analytic pooled HRs, calculated separately in non-smokers and smokers, with their corresponding 95% CIs. The dashed line represents the specific combined risk ratio of smoking status subgroup, and the solid line represents a risk ratio of 1, which is the null hypothesis value. (C) The pooled ratio of OS-HRs reported in smoking status subgroup. (D) The pooled ratio of PFS-HRs reported in smoking status subgroup. Each filled circle indicates the study-specific ratio of HRs. Values less than 1 indicate that the effect of the intervention compared with control is greater for non-smokers than smokers. Size of the circle is proportional to the precision of the estimate. Horizontal lines indicate the 95% CI. The diamond indicates the meta-analytic pooled ratio of HRs, with its corresponding 95% CI. The solid line represents a risk ratio of 1, which is the null hypothesis value.

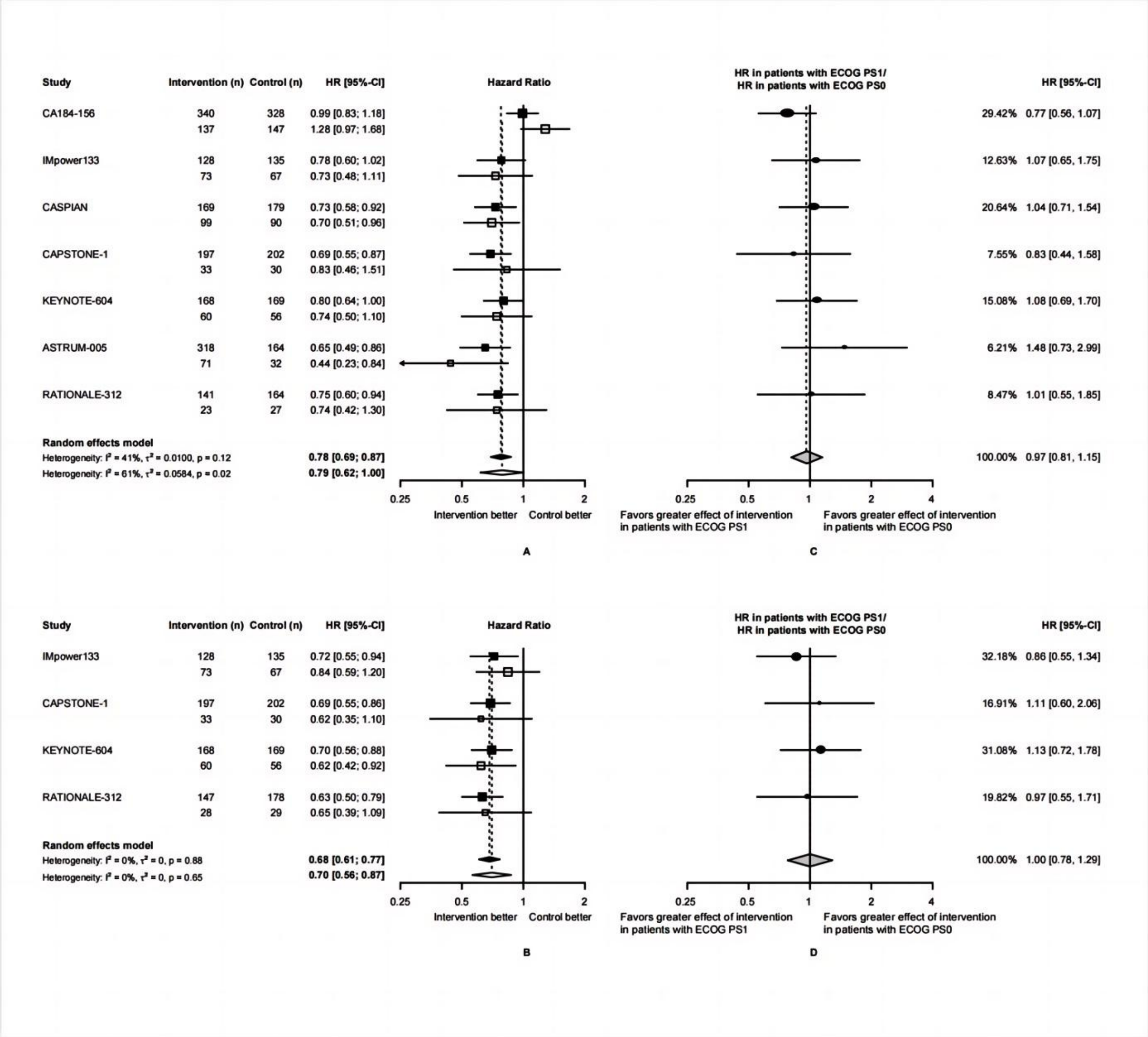

Supplementary Figure 4. Heterogeneity of efficacy between ECOG PS subgroup. Heterogeneity of efficacy between ECOG PS subgroup. (A) The OS-HRs of the intervention and control groups are compared in ECOG PS subgroup. (B) The PFS-HRs of the intervention and control groups are compared in ECOG PS subgroup. Squares indicate study-specific hazard ratios. Values less than 1 indicate intervention is better than control. Size of the square is proportional to the precision of the estimate. Horizontal lines indicate the 95% CI. Diamonds indicate the meta-analytic pooled HRs, calculated separately in patients with ECOG PS 1 and patients with ECOG PS 0, with their corresponding 95% CIs. The dashed line represents the specific combined risk ratio of ECOG PS subgroup, and the solid line represents a risk ratio of 1, which is the null hypothesis value. (C) The pooled ratio of OS-HRs reported in ECOG PS subgroup. (D) The pooled ratio of PFS-HRs reported in ECOG PS subgroup. Each filled circle indicates the study-specific ratio of HRs. Values less than 1 indicate that the effect of the intervention compared with control is greater for patients with ECOG PS 1 than patients with ECOG PS 0. Values of 1 indicate that there was no significant efficacy difference in the intervention group between ECOG PS 0 patients and ECOG PS 1 patients compared with the control group. Size of the circle is proportional to the precision of the estimate. Horizontal lines indicate the 95% CI. The diamond indicates the meta-analytic pooled ratio of HRs, with its corresponding 95% CI. The solid line represents a risk ratio of 1, which is the null hypothesis value.

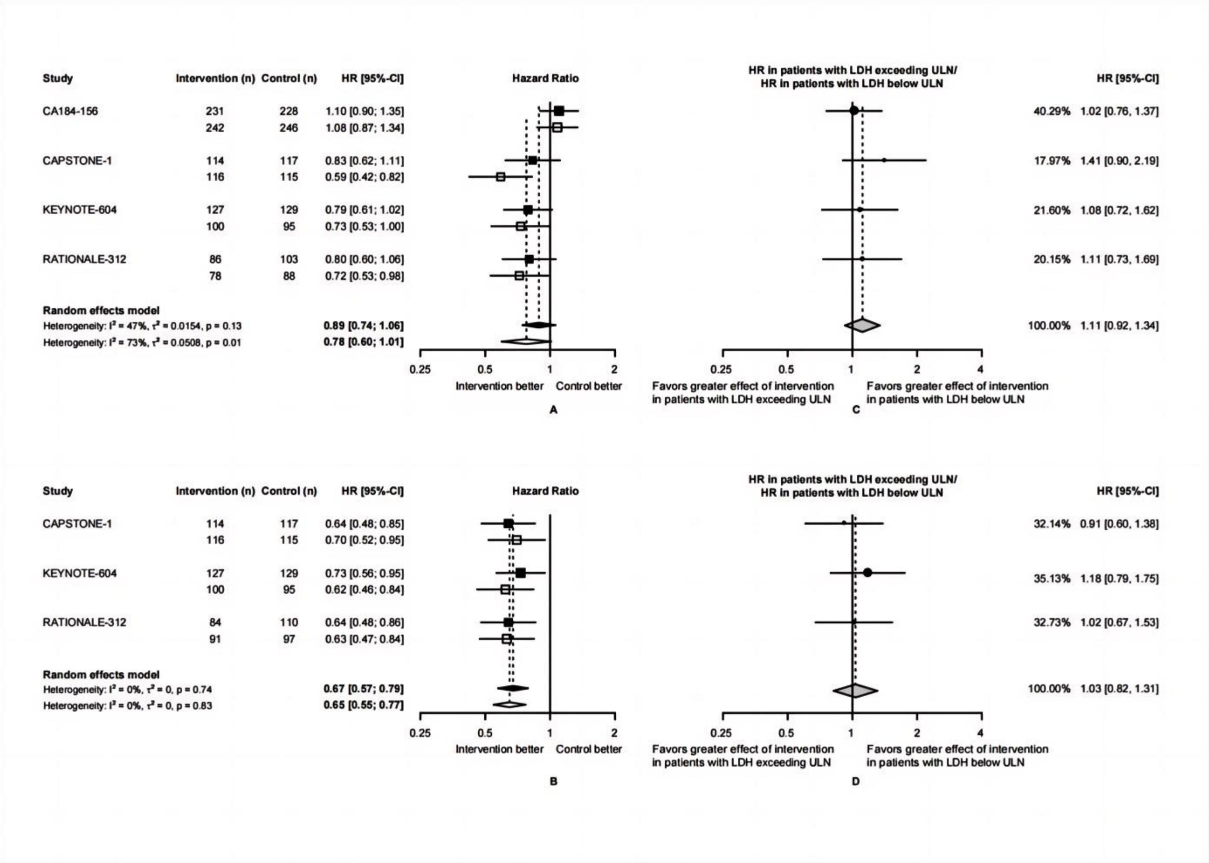

Supplementary Figure 5. Heterogeneity of efficacy between LDH level subgroup. (A) The OS-HRs of the intervention and control groups are compared in LDH level subgroup. (B) The PFS-HRs of the intervention and control groups are compared in LDH level subgroup. Squares indicate study-specific hazard ratios. Values less than 1 indicate intervention is better than control. Size of the square is proportional to the precision of the estimate. Horizontal lines indicate the 95% CI. Diamonds indicate the meta-analytic pooled HRs, calculated separately in patients with LDH≥ULN and patients with LDH<ULN, with their corresponding 95% CIs. The dashed line represents the specific combined risk ratio of LDH level subgroup, and the solid line represents a risk ratio of 1, which is the null hypothesis value. (C) The pooled ratio of OS-HRs reported in LDH level subgroup. (D) The pooled ratio of PFS-HRs reported in LDH level subgroup. Each filled circle indicates the study-specific ratio of HRs. Values more than 1 indicate that the effect of the intervention compared with control is greater for patients with LDH<ULN than patients with LDH≥ULN. Size of the circle is proportional to the precision of the estimate. Horizontal lines indicate the 95% CI. The diamond indicates the meta-analytic pooled ratio of HRs, with its corresponding 95% CI. The solid line represents a risk ratio of 1, which is the null hypothesis value.

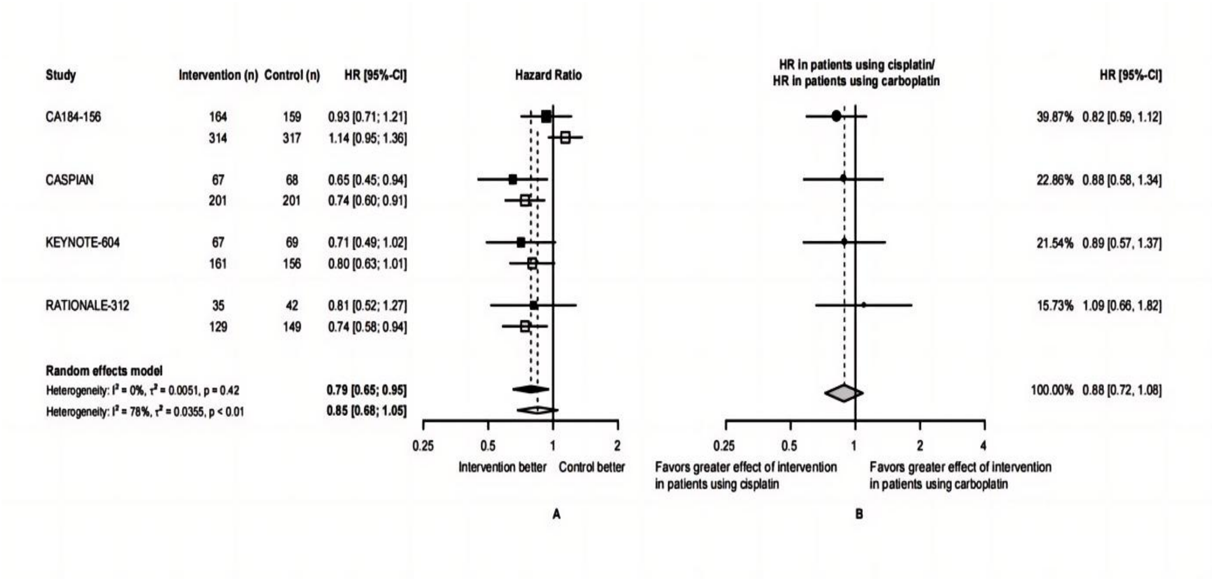

Supplementary Figure 6. Heterogeneity of efficacy between platinum salt subgroup. Heterogeneity of efficacy between platinum salt subgroup. (A) The OS-HRs of the intervention and control groups are compared in platinum salt subgroup. Squares indicate study-specific hazard ratios. Values less than 1 indicate intervention is better than control. Size of the square is proportional to the precision of the estimate. Horizontal lines indicate the 95% CI. Diamonds indicate the meta-analytic pooled HRs, calculated separately in patients using etoposide-cisplatin and patients using etoposide-carboplatin, with their corresponding 95% CIs. The dashed line represents the specific combined risk ratio of platinum salt subgroup, and the solid line represents a risk ratio of 1, which is the null hypothesis value. (B) The pooled ratio of OS-HRs reported in platinum salt subgroup. Values less than 1 indicate that the effect of the intervention compared with control is greater for patients using etoposide-cisplatin than patients using etoposide-carboplatin. Size of the circle is proportional to the precision of the estimate. Horizontal lines indicate the 95% CI. The diamond indicates the meta-analytic pooled ratio of HRs, with its corresponding 95% CI. The solid line represents a risk ratio of 1, which is the null hypothesis value.

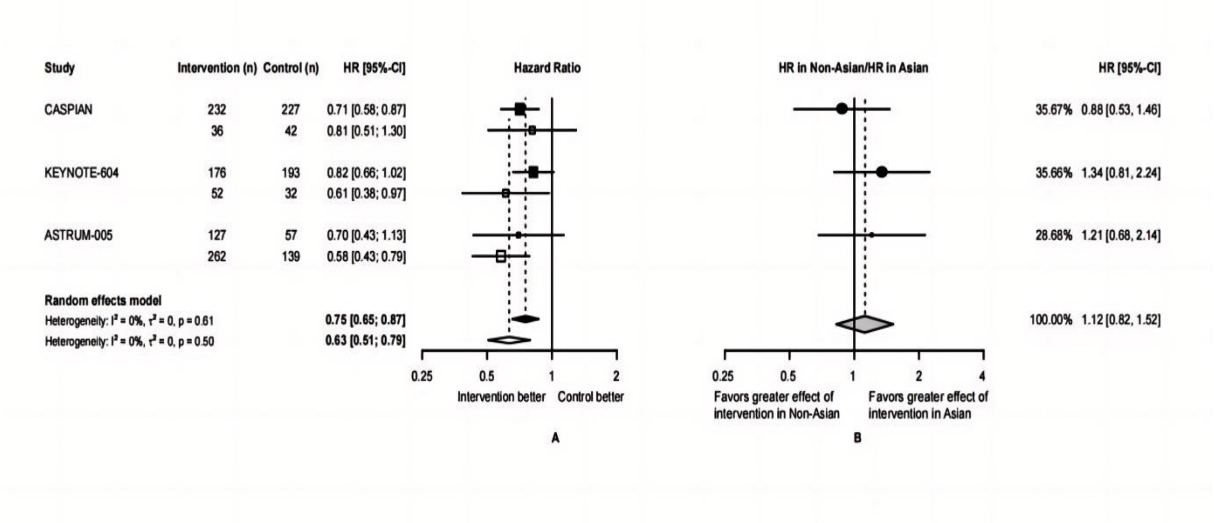

Supplementary Figure 7. Heterogeneity of efficacy between race subgroup. (A) The OS-HRs of the intervention and control groups are compared in race subgroup. Squares indicate study-specific hazard ratios. Values less than 1 indicate intervention is better than control. Size of the square is proportional to the precision of the estimate. Horizontal lines indicate the 95% CI. Diamonds indicate the meta-analytic pooled HRs, calculated separately in non-Asian and Asian, with their corresponding 95% CIs. The dashed line represents the specific combined risk ratio of race subgroup, and the solid line represents a risk ratio of 1, which is the null hypothesis value. (B) The pooled ratio of OS-HRs reported in race

subgroup. Values more than 1 indicate that the effect of the intervention compared with control is greater for Asian than non-Asian. Size of the circle is proportional to the precision of the estimate. Horizontal lines indicate the 95% CI. The diamond indicates the meta-analytic pooled ratio of HRs, with its corresponding 95% CI. The solid line represents a risk ratio of 1, which is the null hypothesis value.

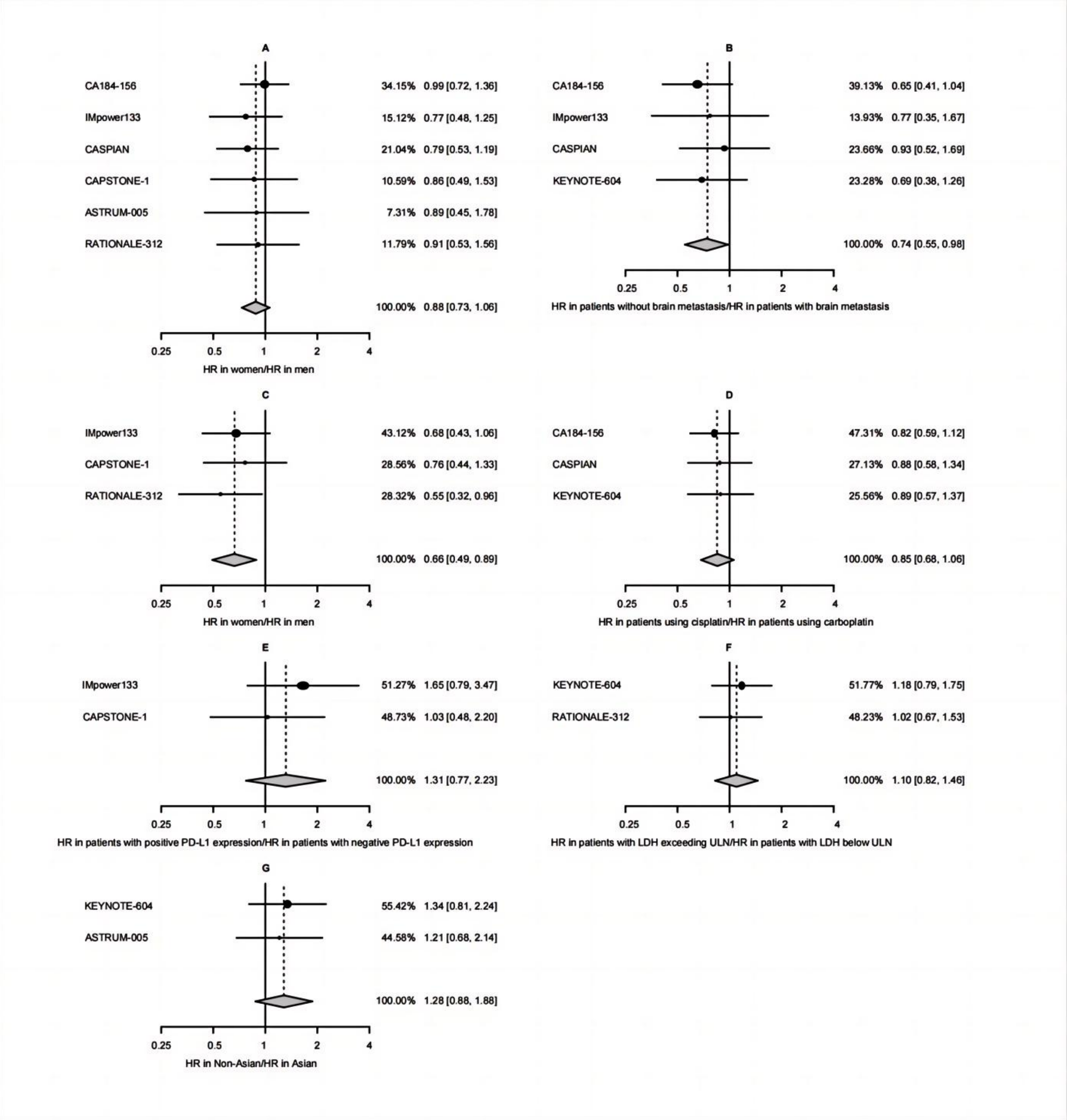

Supplementary Figure 8. Sensitivity analysis. (A) Sensitivity analysis of OS-HR for gender subgroup after removal of KEYNOTE-604 trial; (B) Sensitivity analysis of OS-HR for brain metastases subgroup after removal of ASTRUM-005 trial; (C) Sensitivity analysis of PFS-HR for gender subgroup after removal of KEYNOTE-604 trial; (D) Sensitivity analysis of OS-HR for race subgroup after removal of RATIONALE-312 trial; (E) Sensitivity analysis of PFS-HR for PD-L1 expression level subgroup after removal of KEYNOTE-604 trial; (F) Sensitivity analysis of PFS-HR for LDH level subgroup after removal of CAPSTONE-1 trial; (G) Sensitivity analysis of OS-HR for race subgroup after removal of CASPIAN trial.

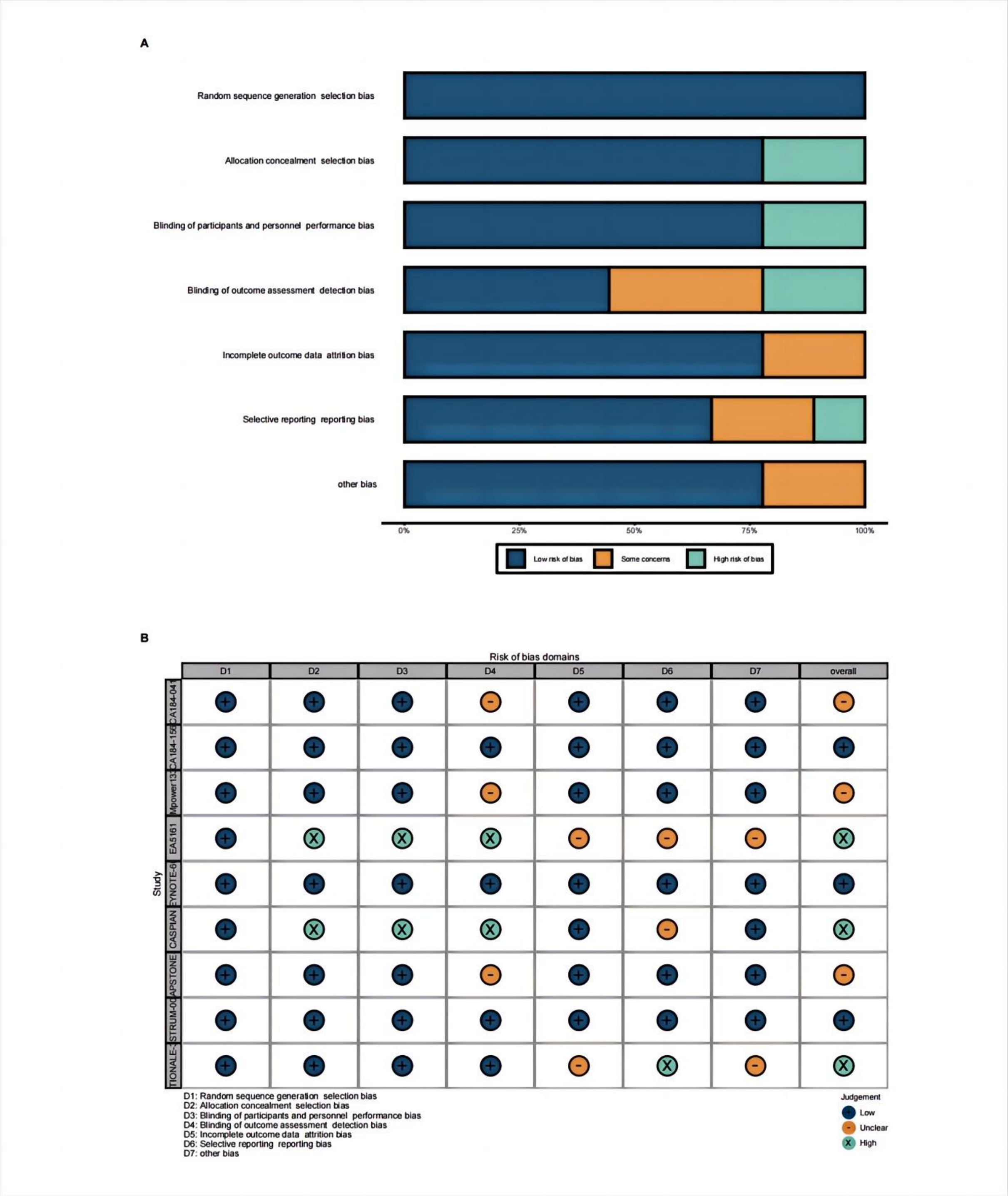

Supplementary Figure 9. Risks of bias for included trials. (A) Risk of bias graph: review authors' judgements about each risk of bias item presented as percentages across all included studies; (B) Risk of bias summary: review authors' judgements about each risk of bias item for each included study.

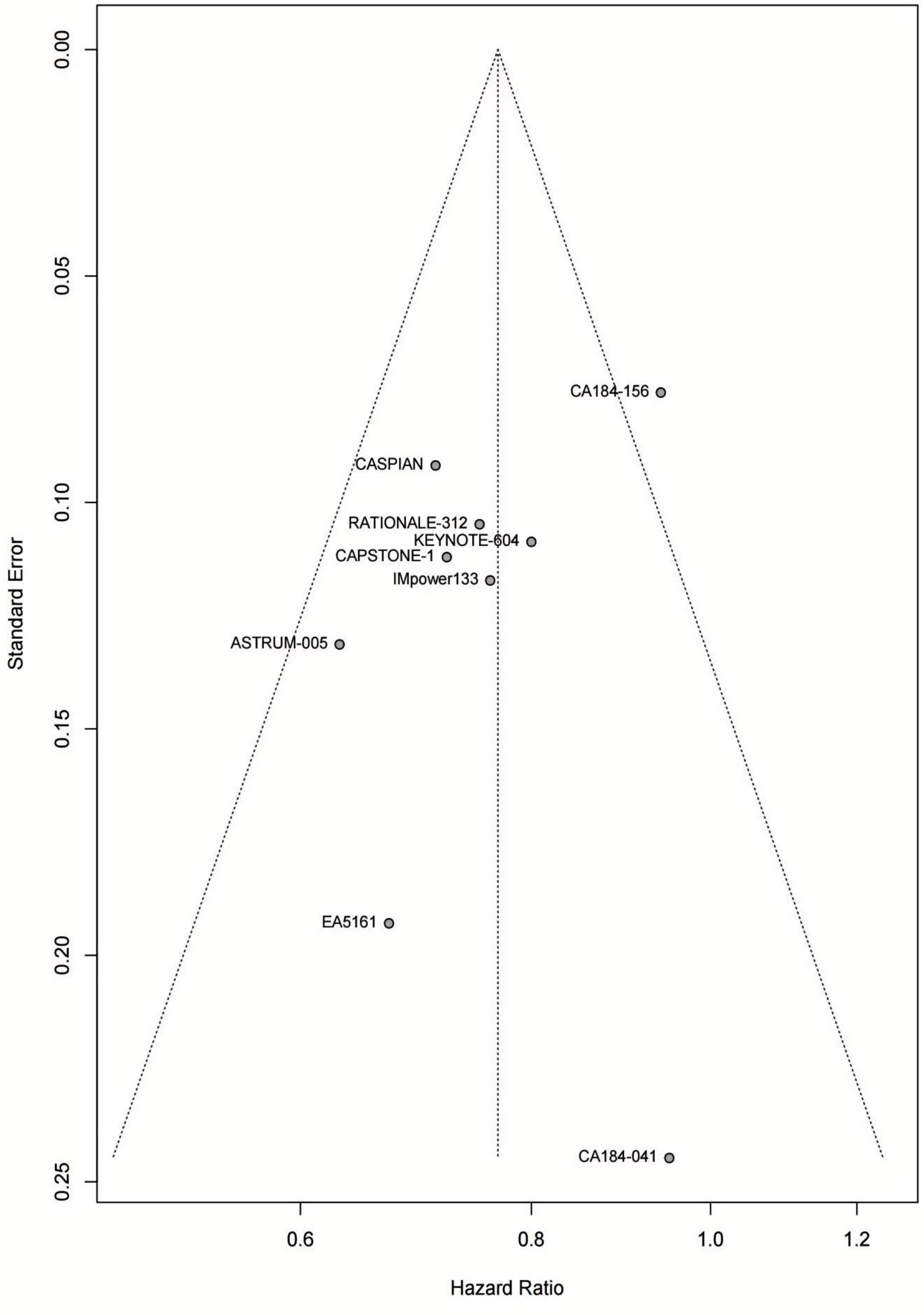

Supplementary Figure 10. Publication bias for included trials.

PRISMA 2020 Checklist

Section and Topic	Item #	Checklist item	Location where item is reported
**TITLE**			
Title	1	Identify the report as a systematic review.	P1
**ABSTRACT**			
Abstract	2	See the PRISMA 2020 for Abstracts checklist.	P1
**INTRODUCTION**			
Rationale	3	Describe the rationale for the review in the context of existing knowledge.	P1-2
Objectives	4	Provide an explicit statement of the objective(s) or question(s) the review addresses.	P2
**METHODS**			
Eligibility criteria	5	Specify the inclusion and exclusion criteria for the review and how studies were grouped for the syntheses.	P3
Information sources	6	Specify all databases, registers, websites, organisations, reference lists and other sources searched or consulted to identify studies. Specify the date when each source was last searched or consulted.	P2-3
Search strategy	7	Present the full search strategies for all databases, registers and websites, including any filters and limits used.	Supplementary Table 1
Selection process	8	Specify the methods used to decide whether a study met the inclusion criteria of the review, including how many reviewers screened each record and each report retrieved, whether they worked independently, and if applicable, details of automation tools used in the process.	P3
Data collection process	9	Specify the methods used to collect data from reports, including how many reviewers collected data from each report, whether they worked independently, any processes for obtaining or confirming data from study investigators, and if applicable, details of automation tools used in the process.	P2-3
Data items	10a	List and define all outcomes for which data were sought. Specify whether all results that were compatible with each outcome domain in each study were sought (e.g. for all measures, time points, analyses), and if not, the methods used to decide which results to collect.	P3
	10b	List and define all other variables for which data were sought (e.g. participant and intervention characteristics, funding sources). Describe any assumptions made about any missing or unclear information.	P3
Study risk of bias assessment	11	Specify the methods used to assess risk of bias in the included studies, including details of the tool(s) used, how many reviewers assessed each study and whether they worked independently, and if applicable, details of automation tools used in the process.	P3
Effect measures	12	Specify for each outcome the effect measure(s) (e.g. risk ratio, mean difference) used in the synthesis or presentation of results.	P3
Synthesis methods	13a	Describe the processes used to decide which studies were eligible for each synthesis (e.g. tabulating the study intervention characteristics and comparing against the planned groups for each synthesis (item #5)).	P3
	13b	Describe any methods required to prepare the data for presentation or synthesis, such as handling of missing summary statistics, or data conversions.	P3
	13c	Describe any methods used to tabulate or visually display results of individual studies and syntheses.	P3
	13d	Describe any methods used to synthesize results and provide a rationale for the choice(s). If meta-analysis was performed, describe the model(s), method(s) to identify the presence and extent of statistical heterogeneity, and software package(s) used.	P3
	13e	Describe any methods used to explore possible causes of heterogeneity among study results (e.g. subgroup analysis, meta-regression).	P3
	13f	Describe any sensitivity analyses conducted to assess robustness of the synthesized results.	P3
Reporting bias assessment	14	Describe any methods used to assess risk of bias due to missing results in a synthesis (arising from reporting biases).	P3
Certainty	15	Describe any methods used to assess certainty (or confidence) in the body of evidence for an outcome.	P3

PRISMA 2020 Checklist

Section and Topic	Item #	Checklist item	Location where item is reported
assessment			
**RESULTS**			
Study selection	16a	Describe the results of the search and selection process, from the number of records identified in the search to the number of studies included in the review, ideally using a flow diagram.	Figure 1
	16b	Cite studies that might appear to meet the inclusion criteria, but which were excluded, and explain why they were excluded.	Figure 1
Study characteristics	17	Cite each included study and present its characteristics.	Table 1
Risk of bias in studies	18	Present assessments of risk of bias for each included study.	Supplementary Figure 9
Results of individual studies	19	For all outcomes, present, for each study: (a) summary statistics for each group (where appropriate) and (b) an effect estimate and its precision (e.g. confidence/credible interval), ideally using structured tables or plots.	P4-5
Results of syntheses	20a	For each synthesis, briefly summarise the characteristics and risk of bias among contributing studies.	P5
	20b	Present results of all statistical syntheses conducted. If meta-analysis was done, present for each the summary estimate and its precision (e.g. confidence/credible interval) and measures of statistical heterogeneity. If comparing groups, describe the direction of the effect.	P4-5
	20c	Present results of all investigations of possible causes of heterogeneity among study results.	P5
	20d	Present results of all sensitivity analyses conducted to assess the robustness of the synthesized results.	P5
Reporting biases	21	Present assessments of risk of bias due to missing results (arising from reporting biases) for each synthesis assessed.	Supplementary Figure 10
Certainty of evidence	22	Present assessments of certainty (or confidence) in the body of evidence for each outcome assessed.	P5
**DISCUSSION**			
Discussion	23a	Provide a general interpretation of the results in the context of other evidence.	P5-8
	23b	Discuss any limitations of the evidence included in the review.	P8
	23c	Discuss any limitations of the review processes used.	P8
	23d	Discuss implications of the results for practice, policy, and future research.	P8
**OTHER INFORMATION**			
Registration and protocol	24a	Provide registration information for the review, including register name and registration number, or state that the review was not registered.	P14
	24b	Indicate where the review protocol can be accessed, or state that a protocol was not prepared.	P14
	24c	Describe and explain any amendments to information provided at registration or in the protocol.	P14
Support	25	Describe sources of financial or non-financial support for the review, and the role of the funders or sponsors in the review.	P14
Competing interests	26	Declare any competing interests of review authors.	P14
Availability of data, code and other materials	27	Report which of the following are publicly available and where they can be found: template data collection forms; data extracted from included studies; data used for all analyses; analytic code; any other materials used in the review.	P14
